# Critical assessment of human metabolic pathway databases: a stepping stone for future integration

**DOI:** 10.1186/1752-0509-5-165

**Published:** 2011-10-14

**Authors:** Miranda D Stobbe, Sander M Houten, Gerbert A Jansen, Antoine HC van Kampen, Perry D Moerland

**Affiliations:** 1 Bioinformatics Laboratory, Academic Medical Center, University of Amsterdam, PO Box 22700, 1100 DE, Amsterdam, the Netherlands; 2 Biosystems Data Analysis, Swammerdam Institute for Life Sciences, University of Amsterdam, Science Park 904, 1098 XH, Amsterdam, the Netherlands; 3 Netherlands Bioinformatics Centre, Geert Grooteplein 28, 6525 GA, Nijmegen, the Netherlands; 4 Netherlands Consortium for Systems Biology, University of Amsterdam, PO Box 94215, 1090 GE, Amsterdam, the Netherlands; 5 Department of Clinical Chemistry, Laboratory Genetic Metabolic Diseases, Academic Medical Center, University of Amsterdam, PO Box 22700, 1100 DE, Amsterdam, the Netherlands; 6 Department of Pediatrics, Emma Children's Hospital, Academic Medical Center, University of Amsterdam, PO Box 22700, 1100 DE, Amsterdam, the Netherlands

## Abstract

**Background:**

Multiple pathway databases are available that describe the human metabolic network and have proven their usefulness in many applications, ranging from the analysis and interpretation of high-throughput data to their use as a reference repository. However, so far the various human metabolic networks described by these databases have not been systematically compared and contrasted, nor has the extent to which they differ been quantified. For a researcher using these databases for particular analyses of human metabolism, it is crucial to know the extent of the differences in content and their underlying causes. Moreover, the outcomes of such a comparison are important for ongoing integration efforts.

**Results:**

We compared the genes, EC numbers and reactions of five frequently used human metabolic pathway databases. The overlap is surprisingly low, especially on reaction level, where the databases agree on 3% of the 6968 reactions they have combined. Even for the well-established tricarboxylic acid cycle the databases agree on only 5 out of the 30 reactions in total. We identified the main causes for the lack of overlap. Importantly, the databases are partly complementary. Other explanations include the number of steps a conversion is described in and the number of possible alternative substrates listed. Missing metabolite identifiers and ambiguous names for metabolites also affect the comparison.

**Conclusions:**

Our results show that each of the five networks compared provides us with a valuable piece of the puzzle of the complete reconstruction of the human metabolic network. To enable integration of the networks, next to a need for standardizing the metabolite names and identifiers, the conceptual differences between the databases should be resolved. Considerable manual intervention is required to reach the ultimate goal of a unified and biologically accurate model for studying the systems biology of human metabolism. Our comparison provides a stepping stone for such an endeavor.

## Background

A detailed description of the human metabolic network is essential for a better understanding of human health and disease [1]. Several of the most prevalent diseases in modern societies, such as cardiovascular disease, diabetes, and obesity have a strong metabolic component. These multifactorial diseases involve hundreds of genes and many developmental and environmental factors. Therefore, network-based approaches are needed to uncover the parts of the molecular mechanisms perturbed by disease [2] and to identify possible drug targets. For example, metabolic networks are nowadays routinely used for the systems-level interpretation of high-throughput data, such as microarray gene expression profiles [3,4].

Over the past fifteen years several groups have constructed high-quality human (metabolic) pathway databases that can be used in this endeavor [5-11]. One of the first pathway databases was the Kyoto Encyclopedia of Genes and Genomes (KEGG) database [8] that was initiated to depart from the existing gene catalogs to pathways. Another example is Reactome [6], which has as one of its main goals to serve as a knowledgebase that describes human biological processes and can be used for computational analyses. The first fully compartmentalized, genome-scale *in silico *model of the human metabolic network is Homo sapiens Recon 1 [7]. This model forms a stepping stone for modeling human metabolic phenotypes.

The various pathway databases available differ in a number of ways and all have their own strengths and weaknesses. For example, they have different solutions for technical issues such as how the data is presented to the user, how one can query the database [12,13], and the exchange formats provided [12,14,15]. Several initiatives, such as BioWarehouse [16] and Pathway Commons [17], have used a data warehouse approach to resolve these differences. By bringing multiple databases under one roof, a data warehouse can be used as "one-stop shop" for answering most of the questions that the source databases can handle, but via a uniform interface [18]. Another type of difference is that the conceptualizations used vary, for example, with respect to the definition of a pathway [12,19]. Furthermore, different databases have taken different approaches in the reconstruction process of the human metabolic network. The reconstruction of the Edinburgh Human Metabolic Network (EHMN) [9], for example, is based on a genome-scale approach using genome annotation as a starting point. Reactome on the other hand takes an incremental approach, regularly adding new parts to its network, and with reactions as basic units [6]. Also the manner and level of curation may differ per database. For instance, Recon 1 is completely manually curated using evidence from literature and then fine-tuned and validated by simulating 288 known metabolic functions *in silico*. Most of the initial content of HumanCyc [11] was automatically derived from both genome annotation and MetaCyc, a multiorganism curated metabolic pathway database, and only curated to a limited extent. Further manual curation of HumanCyc resumed in 2009. Finally, in the reconstruction process evidence from literature may be interpreted differently by curators [1].

It may be apparent that the described differences will have an effect on the metabolic networks defined by the databases. However, so far the various metabolic networks available have not been systematically compared, nor has the extent to which they differ been quantified. For a researcher, *e.g.*, a biomedical scientist who wants to use these databases as a reference repository or a bioinformatician who wants to perform a systems-level analysis of human metabolism, it is crucial to know the extent of the differences in content as well as their underlying causes. The choice for a particular database may, for example, influence the outcome of a computational analysis, as evidenced by diverging results for methods that were applied to multiple metabolic pathway databases [19-22]. Moreover, the sheer variety of metabolic pathway databases is unsatisfactory and their integration is desired. This has been recognized by several groups and integration initiatives are currently ongoing for various organisms [23]. This has already led to the publication of consensus metabolic networks for *S. cerevisiae *[24] and for the human pathogen *S. typhimurium *[25]. The results of a systematic comparison, including the reasons for the differences, can be used as a stepping stone for the reconciliation of human metabolic networks.

We performed a systematic comparison of five frequently used databases, each of which is based on a different approach towards reconstructing the human metabolic network and built by an independent research group: EHMN, Homo sapiens Recon 1 (referred to as BiGG in the rest of the paper), HumanCyc, and the metabolic subsets of KEGG and Reactome. We compared the metabolic reactions, Enzyme Commission (EC) numbers, enzyme encoding genes as well as combinations of these three elements across the five selected databases. We provide an overall analysis, but also compare the tricarboxylic acid (TCA) cycle separately to see in how far the databases agree on this classical metabolic pathway. Our comparison allows us to identify the parts the databases agree on and at the same time to reveal conflicting information. Moreover, current reconstructions of the human metabolic network are work in progress and, therefore, still contain gaps as evidenced by the regular updates of the various databases, reported dead-end metabolites (Recon 1) [7], and listed missing genes (HumanCyc). Our comparison provides a valuable source of complementary information that can be used to fill such knowledge gaps.

Our results show a surprisingly limited level of agreement between the five databases and highlight the challenges to be met when integrating their contents into a single metabolic network.

## Results

For each of the five pathway databases, *i.e.*, BiGG, EHMN, HumanCyc, KEGG, and Reactome (Table 1), we retrieved all metabolic reactions with their corresponding genes, EC numbers, and pathways. Data was imported in a relational database. The database content statistics of the five databases (Table 2) already show that there are notable differences in database size.

**Table 1:** Overview of metabolic pathway databases used

Database	Export formats used	**Version**^**a**^	Downloaded from
BiGG	Flat file, SBML	1	http://bigg.ucsd.edu/
EHMN	Excel	2	http://www.ehmn.bioinformatics.ed.ac.uk/
HumanCyc	Flat file	15.0	http://biocyc.org/download.shtml
KEGG	Flat file, KGML	58	ftp://ftp.genome.jp/pub/kegg/
Reactome	MySQL database	36	http://reactome.org/download/index.html

^a^Downloaded in the first week of May 2011

KGML: KEGG Markup Language; SBML: Systems Biology Markup Language.

**Table 2:** Pathway database content statistics

	Number of			
	
Database	*Genes*	*EC numbers*	*Metabolites*	*Reactions*
BiGG	1496	645	1485	2617
EHMN	2517	940	2676	3893
HumanCyc	3586	1215	1681	1785
KEGG	1535	726	1553	1635
Reactome	1159	356	984	1175

Genes: counts are based on the internal database identifiers and including genes encoding for a component of a protein complex as separate entities. EC numbers: only fully specified EC numbers are counted. Metabolites: counts are based on the internal database identifiers and including instances of metabolite classes for HumanCyc and members of sets for Reactome. Reactions: if reactions only differ in direction and/or compartments they are counted as one.

For each comparison we calculated the *consensus*, defined as the overlap between the databases as a percentage of their union

$$\text{consensus }=\frac{\left|C_{\text{BiGG}}\cap C_{\text{EHMN}}\cap C_{\text{HumanCyc}}\cap C_{\text{KEGG}}\cap C_{\text{Reactome}}\right|}{|C_{\text{BiGG}}\cup C_{\text{EHMN}}\cup C_{\text{HumanCyc}}\cup C_{\text{KEGG}}\cup C_{\text{Reactome}}|\times \mathrm{100\%}}$$

where *C *is the set of entities (genes, EC numbers, metabolites, reactions) under consideration. The consensus is constrained by the smallest database for a specific entity, which is in all cases Reactome. Therefore, we also calculated a score that is less sensitive to these differences in database size, the *majority score*, defined as the number of entities that occurs in at least three out of the five pathway databases as a percentage of their union. To limit the impact of out-of-date identifiers and EC numbers on our comparison, the ones that had been transferred were replaced by their new ID/EC number and otherwise they were not used in the comparison (Additional file 1).

### Comparison: genes

**Figure 1:**
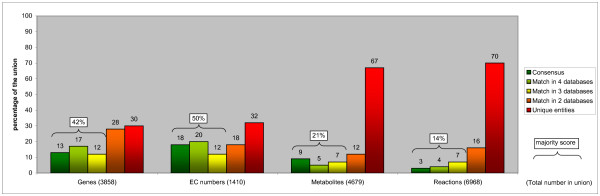
**Overlap between the five metabolic pathway databases for the global comparison**. The dark green bars give the percentage of entities (genes, EC numbers, metabolites and reactions) that are part of the consensus. The majority score is given by the combined percentages of the dark green, light green (4 out of 5 databases agree) and yellow (3 out of 5) bars. The orange bars indicate the percentage of entities that can only be found in 2 databases. The percentage of unique entities is indicated by the red bars. In matching the reactions we did not take into account e^-^, H^+ ^and H_2_O.

Although some reactions in the metabolic network may take place spontaneously, most reactions are catalyzed by an enzyme. In the first comparison we, therefore, investigated the genes encoding for these enzymes by comparing their Entrez Gene IDs. The consensus on gene level is only 13% of the 3858 Entrez Gene IDs contained in the union of all five databases (Table 3, Figure 1). The majority score shows that only 42% of all genes can be found in at least three databases. There are 1139 genes that are present in only one of the databases, representing 30% of the total.

**Table 3:** Statistics of the pathway database comparison

	Number of (percentage of union)				
	
	*Genes*	*EC numbers*	*Metabolites*	*Reactions*	***Reactions (ignoring e***^-^ ***, H***^***+***^ ***, H***_***2***_ ***O)***
union	3858	1410	4679	7758	6968
consensus	510 (13%)	259 (18%)	400 (9%)	101 (1%)	199 (3%)
majority score	1636 (42%)	709 (50%)	967 (21%)	732 (9%)	1004 (14%)

**Database**	**Unique per database (percentage of union)**				

BiGG	45 (1%)	48 (3%)	528 (11%)	1441 (19%)	1250 (18%)
EHMN	128 (3%)	82 (6%)	1184 (25%)	2120 (27%)	1832 (26%)
HumanCyc	759 (20%)	294 (21%)	739 (16%)	1032 (13%)	905 (13%)
KEGG	63 (2%)	13 (1%)	282 (6%)	414 (5%)	348 (5%)
Reactome	144 (4%)	11 (1%)	406 (9%)	601 (8%)	539 (8%)

Total	1139 (30%)	448 (32%)	3139 (67%)	5608 (72%)	4874 (70%)

Genes: Entrez Gene IDs, including genes encoding for a component of a protein complex as separate entities. EC numbers: only fully specified EC numbers. Metabolites: if two metabolites of one database both match the same metabolite in another database this is counted as one match. The first 'Reactions' column: all reactions are considered. The second 'Reactions' column: reactions were not required to match on e^-^, H^+ ^and/or H_2_O.

We compared the gene ontology (GO) annotation of the 510 genes in the consensus on gene level versus the union of the remaining genes using FatiGO [26] to gain a better understanding of the biological processes the consensus genes are involved in. The set of consensus genes is significantly enriched (adjusted P < 0.01) for processes related to the generation of precursor metabolites and energy, nucleotide metabolism, alcohol metabolism, and cofactor metabolism (Additional file 2).

### Comparison: EC numbers

The Nomenclature Committee of the International Union of Biochemistry and Molecular Biology (NC-IUBMB) classifies and names enzymes according to the reaction they catalyze [27]. EC numbers are used as the vocabulary to describe this classification. An EC number consists of four numbers. The first three indicate increasingly narrower classes of enzymatic functions. The fourth number serves as a serial number and defines the substrate specificity of the enzyme [28]. Comparing EC numbers on the basis of only the first three numbers (ignoring the last) thus gives a global indication whether the databases agree on the types of enzymatic functions involved in the human metabolic network. There are 164 unique entries in the union of all databases with a consensus of 51%. For the remaining 49% the five databases do not agree. For example, the group of peptidases present in the union of the five databases is not part of the consensus.

If we compare complete EC numbers, thus taking into account the serial number that represents substrate specificity, the consensus decreases to 18% of the 1410 EC numbers contained in the union (Table 3, Figure 1). Of the total set of EC numbers 32% can only be found in a single database, primarily HumanCyc.

### Comparison: metabolites

Agreement on the metabolites that are part of the metabolic network is a prerequisite for consensus between databases on reaction level. Metabolites were matched based on the KEGG Compound ID, if available for both metabolites. If the KEGG Compound ID was absent, metabolites were matched on one of the other four available metabolite identifiers (KEGG Glycan, ChEBI, PubChem Compound or CAS) or on metabolite name, provided that also the chemical formula matched. The consensus for the metabolites is only 9% of the 4679 metabolites contained in the union (Table 3, Figure 1). The majority score equals 21% of the metabolites.

### Comparison: reactions

Reactions were considered to be the same if all substrates and products matched (see above). As expected, given the outcome of the metabolite comparison, the number of reactions included in all five databases is small: consensus on reaction level equals 1% of 7758 reactions in the union of all five databases (Table 3).

Reactions are not always balanced, especially with respect to electrons (e^-^), protons (H^+^) and water (H_2_O) [29]. Therefore, we performed a second comparison where reactions were not required to match with respect to these three metabolites. The number of reactions in the consensus nearly doubled to 199 reactions, corresponding to 3% of the 6968 reactions in total. The majority score for this reaction comparison equals 14% (Table 3, Figure 1). Around one third of the 199 consensus reactions are part of nucleotide metabolism or cofactors and vitamins metabolism (Additional file 3). This is in line with the results of the functional enrichment analyses of the consensus genes.

We compared a relatively large number of pathway databases, each restricting the consensus, which partly explains the small overlap. If we only compare pairs of databases, the overlap on reaction level increases substantially. The consensus of two databases ranges from 11%, when comparing EHMN and Reactome, to as much as 28% when comparing EHMN and KEGG (Additional file 4). The pairwise comparisons on gene, EC number, and metabolite level also show a substantial increase in overlap.

### Comparison: combinations

So far, we only compared the databases on a single level. We also investigated the consensus on two levels by requiring the gene and the complete EC number to match. For 35% of the 510 genes in the consensus, the five databases also agree on all EC number(s) connected to a gene. For 63% of the consensus genes the databases agree on at least one EC number. The mismatches suggest that the databases do not fully agree on the enzymatic activities that gene products can have.

If we require an exact match on all three levels - EC number, gene, and reaction - then the five pathway databases agree on the genes and EC numbers of 85 of the 199 reactions in the consensus (when not taking into account e^-^, H^+ ^and H_2_O). For 44 reactions the databases agree only on the EC number and for 25 reactions only on the genes. For 24 consensus reactions there is not a single EC number the databases agree on and not a single gene for 9 reactions. The main reason (57 reactions) that there is no agreement on all genes is because one or more of the databases links additional genes to the reaction in comparison to the other databases. See Additional file 3 for a detailed summary of the consensus reactions with their associated EC numbers, genes, and pathways.

### Comparison: TCA cycle

**Figure 2:**
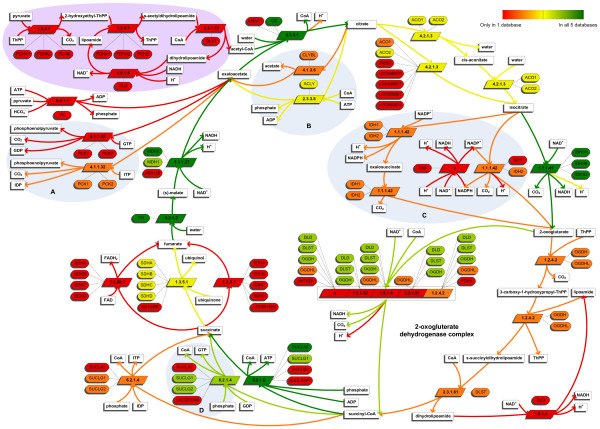
**Comparison of the TCA cycle in five metabolic pathway databases**. Map illustrating the (lack of) consensus for the TCA cycle. Metabolites are represented by rectangles, genes by rounded rectangles, and EC numbers by parallelograms. Color indicates how many of the five databases agree on a specific entity (gene, EC number, reaction). We first matched reactions based on their metabolites. Genes and EC numbers were matched within matching reactions. Color of an arrow indicates the number of databases that agree upon an entire reaction, *i.e.*, all its metabolites (except H^+ ^which was matched separately). 'x' denotes a missing EC number. Areas labeled A-D highlight the reactions that are discussed in the running text as examples of differences caused by a disagreement on pathway definition.

We also analyzed the well-known TCA cycle, already described in 1938 by Krebs [30]. For this pathway we expected a high agreement between the databases. However, also for the TCA cycle the consensus on reaction level is surprisingly low, although higher than what was observed at database level. The databases agree on 5 (17%) of the 30 reactions in total (Table 4 and Figure 2, see Additional files 5 and 6 for a breakdown per database). On gene level the consensus is 36% of 45 genes and on EC number level the consensus is 30% of 20 EC numbers. For the five reactions in the reaction consensus, the databases all agree on the EC number and on at least one gene. Only for two reactions (EC 1.1.1.41, EC 4.2.1.2) they agree on exactly the same set of genes.

**Table 4:** Statistics of the comparison of the TCA cycle

	Number of (percentage of union)			
	
	*Genes*	*EC numbers*	*Metabolites*	*Reactions*
Union	45	20	41	30
Consensus	16 (36%)	6 (30%)	18 (44%)	5 (17%)
Majority	23 (51%)	11 (55%)	25 (61%)	12 (40%)

Genes: including genes encoding for a component of a protein complex as separate entities. EC numbers: fully specified EC numbers. Metabolites: several metabolites were matched manually (see Materials and Methods). Reactions: reactions where not required to match on H^+^.

## Analysis of differences between databases

The above results show that consensus between the five databases is low on all levels compared. This is most pronounced for the reactions. First, we use the TCA cycle to illustrate a number of reasons for these differences. Next, we describe how this translates to the comparison at database level.

### TCA Cycle

In what follows we present the main, sometimes overlapping, causes for lack of consensus at the reaction level: (*i*) disagreement on pathway definition, (*ii*) difference in number of intermediate steps, (*iii*) a different number of possible alternative substrates. In addition, it is difficult to determine when databases refer to the same metabolite. Missing and out-of-date gene identifiers also hinder the comparison. Since genes and EC numbers are tightly linked to reactions, most differences on these two levels are caused by differences on the reaction level. We conclude with additional causes for lack of consensus for genes and EC numbers.

#### Pathway definition

The five pathway databases each have their own definition of which reactions are part of the pathway describing the TCA cycle. For example, in KEGG the conversion of pyruvate into acetyl-CoA is included in the TCA Cycle (Figure 2, purple area). In EHMN and BiGG this conversion is part of the glycolysis/gluconeogenesis pathway and in the other two databases it is part of a separate pathway. The differences in pathway definition are further illustrated by several reactions that are not in the consensus of the TCA cycle, but are part of the consensus at database level:

A. The reaction transforming oxaloacetate into phosphoenolpyruvate (EC 4.1.1.32 via GTP → GDP, Figure 2). In general, this reaction, although tightly linked to the TCA Cycle, is considered to be part of gluconeogenesis [31]. However, KEGG includes this reaction in the TCA cycle pathway. KEGG and EHMN also mention the same conversion with an alternative cosubstrate (EC 4.1.1.32 via ITP → IDP). The latter reaction is not part of the consensus at database level.

B. The reaction converting citrate back to oxaloacetate (EC 2.3.3.8). This reaction is found in BiGG, EHMN, and KEGG. According to Reactome the reaction belongs to the pathway 'Fatty Acyl-CoA Biosynthesis' and HumanCyc assigns it to 'acetyl-CoA biosynthesis (from citrate)'. Moreover, Reactome also provides evidence that the reaction takes place in the cytosol and not in the mitochondrion where the TCA cycle takes place. Interestingly, BiGG and EHMN also claim that the reaction does not take place in the mitochondrion, but they include it in the TCA cycle nevertheless.

C. The reaction transforming succinyl-CoA into succinate via GDP → GTP (EC 6.2.1.4). This reaction is described in HumanCyc, but was not assigned to any pathway.

D. The interconversion of NAD^+^/NADPH and NADH/NADP^+^. Only Reactome includes this reaction in the TCA Cycle, in the other four databases it is part of pathways related to nicotinate and nicotinamide metabolism.

Differences in pathway definition explain why 14 of the 30 reactions are not in the consensus (Additional file 6).

#### Number of intermediate steps

Another explanation for the differences observed is that the number of intermediate steps used to describe a specific conversion varies. A typical example is the oxidative decarboxylation of 2-oxoglutarate to succinyl-CoA (2-oxoglutarate dehydrogenase complex). KEGG describes this reaction in four steps. In BiGG, HumanCyc and Reactome the entire oxidative decarboxylation is described in a single step. Interestingly, EHMN describes it both in a single step as well as in three steps.

The databases also disagree on the number of steps for describing the conversion of citrate to isocitrate (EC 4.2.1.3). In BiGG and Reactome this is a single step, but it takes two steps in HumanCyc and KEGG with cis-aconitate as intermediate. Indeed, cis-aconitate has been shown to be an intermediate in the conversion of citrate into isocitrate [31,32]. EHMN includes both the single and the two-step variant. Note that there is no automated way in which we could tell whether the difference in the number of steps is because of a difference in the level of detail used to describe a particular conversion or due to a disagreement on the number of steps needed for that conversion. Differences in number of intermediate steps explain 14 mismatches on reaction level.

#### Number of alternative substrates

A third explanation for the observed differences is the variation in the number of possible alternative substrates listed. This is, for example, observed for the type of nucleotide diphosphate as cosubstrate for the conversion of succinyl-CoA to succinate. According to EHMN and KEGG, not only ADP (EC 6.2.1.5) can be used, but also IDP (EC 6.2.1.4). Differences caused by alternative substrates explain six mismatches on reaction level.

#### Establishing identity

The comparison of the metabolites is hindered by the difficulty of determining in an automated way when databases refer to the same compound. For example, we decided for three pairs of metabolites that the databases are referring to the same metabolite, despite that the databases linked different KEGG Compound IDs to these metabolites (see Materials and Methods). The only difference between these pairs, is that one is the enzyme bound form of the metabolite, *e.g.*, lipoamide-E (KEGG Compound ID: C15972), and the other is indicated as being unbound, *e.g.*, lipoamide (KEGG Compound ID: C00248). For a relatively small pathway like the TCA Cycle, such highly similar compounds can be easily identified manually, but on database level this is very challenging.

Also out-of-date and missing identifiers influence the comparison. Five unmatched genes from Reactome had an Entrez Gene ID that had become obsolete and could not be transferred to another entry. For a single gene in HumanCyc there were no gene identifiers available at all.

#### Additional explanations on gene and EC number level

On gene level, ten differences remain that are not caused by differences on the reaction level or out-of-date identifiers. Three genes (ACO1, IREB2, and MDH1) encode for proteins that are not localized in the mitochondrion, according to the UniProt annotation. Since the TCA cycle takes place in the mitochondrion, these may be annotation errors of the pathway databases. In BiGG PDHX encodes for a component of the 2-oxoglutarate complex, but according to Entrez Gene it encodes for a component of the similar, but different, pyruvate dehydrogenase complex. The gene OGDHL, which is found in three databases, is described by Entrez Gene as 'oxoglutarate dehydrogenase-like', which refers to the OGDH gene that is part of the consensus. For two genes (LOC283398 and SUCLA2P1) in Reactome the RefSeq status is 'inferred', which may be a reason for the other databases to not include these genes. For the other three genes (AMAC1, DHTKD1, MDH1B) there is no clear explanation. Possibly these are incorrectly connected to the reactions of the TCA cycle.

For four EC numbers the differences on reaction level do not explain why they are not part of the consensus. All four are assigned to the reaction converting 2-oxoglutarate to succinyl-CoA by at least one of the databases. Three of these EC numbers belong to the individual components of the complex catalyzing the reaction. BiGG only assigns one (EC 1.2.4.2) of these three to the catalyst, EHMN assigns all three and HumanCyc leaves the EC number blank. According to IUBMB the EC number (EC 1.2.1.52) assigned by Reactome belongs to the enzyme that can catalyze a similar reaction, but with NADP^+^/NADPH as cosubstrates instead of NAD^+^/NADH.

### Database level

The explanations we gave for the lack of consensus in the TCA cycle can be generalized to the comparisons at database level. One exception is the difference in pathway definition, as the subdivision of the network in pathways no longer plays a role in the comparisons on database level. However, a similar effect can be observed due to differences in metabolic network coverage.

#### Metabolic network coverage

All five databases are work in progress and, therefore, do no yet fully cover the complete metabolic network. As the database content statistics (Table 2) show, there are large differences in the number of genes, EC numbers, and reactions contained in each database. On gene and EC number level HumanCyc is largely a superset of the other four databases and contains the highest number of unique entities on these two levels. EHMN has the highest number of metabolites and reactions. This is to a large extent explained by a set of 1100 transport reactions and 1016 reactions in lipid metabolism contained in EHMN, compared to 484 and 211, respectively, in Reactome, for example. In general, the size differences can be partly explained by the different criteria the five databases have for including reactions in their metabolic network. A difference in coverage could also to some extent explain the large percentage of data that is only found in one of the databases. For example, there are 1139 unique genes and 4874 unique reactions (Table 3).

To gain a better understanding of which parts of the metabolic network are only described in a single database, we compared the GO annotation of all 1139 unique genes versus the union of the remaining genes using FatiGO (Additional file 2). The unique genes are significantly enriched for terms related to ion transport, protein metabolism like proteolysis, and to RNA metabolism such as tRNA processing.

For a more in-depth analysis of the coverage of the individual databases, we compared for each database separately its unique genes with the remaining genes contained in the union (Additional file 2). HumanCyc has the largest set of unique genes, which are significantly enriched for terms related to, among others, ion transport, protein metabolic processes like proteolysis, and (t)RNA processing. Enriched terms for Reactome include transport, protein catabolic processes, and regulation of catalytic activity. As metabolic and non-metabolic reactions in Reactome are intertwined, this might be an indication that some non-metabolic reactions are described in the metabolic pathways we selected. EHMN only has few significant terms, which are related to Golgi vesicle transport and budding. EHMN contains the highest number of transport reactions, but 55% of these are not linked to a gene and, therefore, do not influence the GO analysis. BiGG and KEGG contain the lowest number of unique genes and only BiGG has a significantly enriched GO term, namely signal peptide processing.

**Figure 3:**
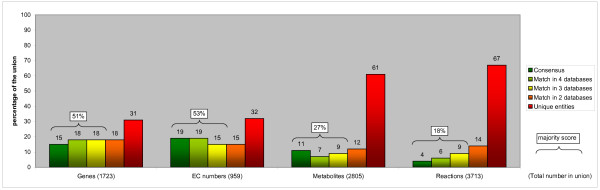
**Overlap between the five metabolic pathway databases for the comparison of the core metabolic processes**. The dark green bars give the percentage of entities (genes, EC numbers, metabolites and reactions) that are part of the consensus in the comparison restricted to the following six categories: amino acid metabolism, carbohydrate metabolism, energy metabolism, lipid metabolism, metabolism of cofactors and vitamins, and nucleotide metabolism. The majority score is given by the combined percentages of the dark green, light green (4 out of 5 databases agree) and yellow (3 out of 5) bars. The orange bars indicate the percentage of entities that can only be found in 2 databases. The percentage of unique entities is indicated by the red bars. In matching the reactions we did not take into account e^-^, H^+ ^and H_2_O.

The GO enrichment analysis shows that the pathway databases are partly complementary and include reactions that are peripheral to metabolism proper, such as ion transport and macromolecular reactions. On the other hand, the genes contained in the majority of the databases compared to the union of the remaining genes, proved to be significantly enriched for many of what one could consider to be core metabolic processes (Additional file 2): nucleotide metabolism, carbohydrate metabolism, lipid metabolism, and generation of energy, among others. One might, therefore, conjecture that the consensus between the databases would significantly increase by restricting the comparison to core metabolic processes. For this purpose, we first grouped the pathways from each of the databases into categories using the KEGG hierarchy as a guideline (see Materials and Methods, Additional file 7). Next, we restricted the comparison to the following six core metabolic categories: amino acid metabolism, carbohydrate metabolism, energy metabolism, lipid metabolism, metabolism of cofactors and vitamins, and nucleotide metabolism. Reactions not part of any pathway could not be assigned to a category and were therefore excluded. Furthermore, we excluded macromolecular reactions, *i.e.*, reactions in which at least one metabolite was labeled as being a protein, for HumanCyc and Reactome. Also transport reactions found in BiGG, EHMN, HumanCyc and Reactome were excluded. The database content statistics for this comparison of core metabolic processes are given in Table 5. The consensus hardly changes for any of the entities compared (Figure 3, Table 6). The majority score for the gene comparison augmented considerably by 9%. For the reaction comparison the majority score increased by only 4%.

**Table 5:** Pathway database content statistics of core metabolic processes

	Number of (percentage of total)			
	
Database	*Genes*	*EC numbers*	*Metabolites*	*Reactions*
BiGG	957 (64%)	558 (87%)	1041 (70%)	1301 (50%)
EHMN	1221 (49%)	707 (75%)	1924 (72%)	2180 (56%)
HumanCyc	832 (23%)	503 (41%)	847 (50%)	815 (46%)
KEGG	1291 (84%)	619 (85%)	1190 (77%)	1308 (80%)
Reactome	413 (36%)	279 (78%)	532 (54%)	511 (43%)

Genes: counts based on the internal database identifiers and including genes encoding for a component of a protein complex as separate entities. EC numbers: only fully specified EC numbers are counted. Metabolites: counts based on the internal database identifiers and including instances of metabolite classes for HumanCyc and members of sets for Reactome. Reactions: if reactions only differ in direction and/or compartments they are counted as one.

**Table 6:** Statistics of the pathway database comparison of core metabolic processes

	Number of (percentage of union)							
	
	*Genes*		*EC numbers*		*Metabolites*		*Reactions*	
union	1723	-55%	959	-32%	2805	-40%	3713	-47%
consensus	264 (15%)	+ 2%	180 (19%)	+ 1%	316 (11%)	+ 2%	144 (4%)	+ 1%
majority score	875 (51%)	+ 9%	508 (53%)	+ 3%	757 (27%)	+ 6%	674 (18%)	+ 4%

**Database**	**Unique per database (percentage of union)**							

BiGG	75 (4%)	+ 3%	63 (7%)	+ 4%	276 (10%)	- 1%	621 (17%)	- 1%
EHMN	186 (11%)	+ 8%	125 (13%)	+ 7%	910 (32%)	+ 7%	1129 (30%)	+ 4%
HumanCyc	70 (4%)	-16%	62 (6%)	-15%	243 (9%)	- 7%	326 (9%)	- 4%
KEGG	180 (10%)	+ 8%	45 (5%)	+ 4%	209 (7%)	+ 1%	274 (7%)	+ 2%
Reactome	19 (1%)	- 3%	8 (1%)	0	80 (3%)	- 6%	154 (4%)	- 4%

**Total**	530 (31%)	+ 1%	303 (32%)	0	1718 (61%)	- 6%	2504 (67%)	- 3%

Genes: Entrez Gene IDs, including genes encoding for a component of a protein complex as separate entities. EC numbers: only fully specified EC numbers. Metabolites: if two metabolites of one database both match the same metabolite in another database this is counted as one match. Reactions: reactions where not required to match on e^-^, H^+ ^and/or H_2_O. Differences with the outcomes of the global comparison (Table 3) are indicated in percentages for each level of comparison.

These results support the conclusion that the networks are partly complementary, but also indicate that there are additional reasons for the lack of overlap, which we will describe below.

#### Number of intermediate steps

In the comparison of the TCA cycle a difference in the number of steps used to describe a specific metabolic conversion could easily be identified manually. On database level, however, this poses a considerable challenge and would require very generic tools for network alignment. One indication that the problem is not restricted to the TCA cycle is given by 64 reactions in BiGG for which the comments in the SBML file indicate that the reaction summarizes a conversion that actually consists of several steps. For example, BiGG describes the breakdown of palmitoyl-CoA to octanoyl-CoA in a single step. However, this is a simplification of four rounds of beta oxidation, each round consisting of four separate reactions. In KEGG the same conversion makes up a large part of the 'fatty acid metabolism' pathway.

#### Number of alternative substrates

The number of reactions linked to one of the 259 consensus EC numbers varies considerably across the databases and equals 411 for Reactome, 441 for HumanCyc, 539 for BiGG, 582 for KEGG, and 942 for EHMN. A possible explanation for a low number of reactions is the use of a single generic reaction to model the broad substrate specificity of an enzyme instead of explicitly describing each specific reaction separately with the same EC number. HumanCyc, for example, uses generic metabolites, such as 'an alcohol', in 24% of the reactions linked to an EC number from the consensus. The high number of reactions in EHMN is at least partly explained by the number of alternative substrates specified. Focusing on lipid metabolism, the median number of reactions per EC number is three for EHMN, while for HumanCyc, for example, the median is one. The effect of alternative substrates has been noticed before [33] in a comparison of all reactions in BRENDA [34], ENZYME [35], and KEGG. Reactions in these databases overlapped for only 21%. Consensus increased to 67% when they included only the main reactions, as defined by IUBMB, of BRENDA and not the reactions derived from these with alternative substrates.

#### Establishing identity

The difficulty of determining when databases refer to the same compound partly explains the lack of overlap on metabolite level and consequently on reaction level. Metabolite identifiers provide a common ground for finding corresponding metabolites in a reliable way, provided the correct identifier was assigned to each metabolite. The only identifier type that is shared among the five databases and that is available for a substantial number of metabolites is the KEGG Compound ID (Additional files 8 and 9). Unfortunately, for 34% (HumanCyc) to 42% (BiGG) of the metabolites included in the pathway databases, except for KEGG, this identifier is missing. In KEGG for 8% of its metabolites the KEGG Glycan ID is provided instead. To increase the number of metabolites for which we could potentially identify corresponding metabolites we also included KEGG Glycan, ChEBI, PubChem and CAS IDs for the comparison. However, 25% (HumanCyc) to 34% (BiGG) of the metabolites included in the pathway databases (except KEGG) were not linked to any of the four metabolite databases (Additional file 9). We, therefore, decided to also match on the metabolite name, which has as disadvantage that there will often be a large number of, possibly ambiguous, synonyms and spelling variants [36]. To restrict the possibility of false positive matches caused by matching on the metabolite name, we also required the chemical formula to match.

Even using this strategy, a large number of metabolites without identifier remains that could not be matched on name, see Additional file 10 for an overview. This overview shows that the majority of these unique metabolites are part of specific metabolic processes, illustrating the different choices made by each of the databases. In EHMN, for example, 60% of the unique metabolites without an identifier are part of lipid metabolism. In BiGG, 55% is found in glycan biosynthesis and metabolism, *e.g. *precursors or degradation products of long unbranched polysaccharides such as chondroitin sulfate, heparin sulfate, or keratan sulfate. Furthermore, in Reactome, 64% of the unique reactants without a metabolite identifier are proteins and complexes directly encoded by the genome and have a UniProt ID instead. In HumanCyc, finally, 55% of the metabolites are part of reactions that have not been assigned to any pathway and which are possibly peripheral to metabolism proper. Restricting the comparison to the core metabolic processes and removing macromolecular reactions from Reactome and HumanCyc, reduced the impact of the mismatches because of missing metabolite identifiers. For BiGG, HumanCyc, and Reactome the percentage of metabolites without an identifier decreased from 34%, 25%, and 31% to 21%, 13% and 10%, respectively (Additional file 11). Since lipid metabolism is part of the core comparison, EHMN is still greatly affected by the lack of identifiers for lipids and misses an identifier for 38% of its metabolites. There is a large variety of lipids, which may explain the lack of identifiers for this type of metabolite.

On gene level the only identifier type shared by all five databases is the Entrez Gene ID (Additional file 8). In total 356 genes do not have an Entrez Gene ID (after removing obsolete IDs) most of which are contained in HumanCyc (327 genes). On the level of EC numbers the five databases combined contain 83 EC numbers that are not fully specified. Moreover, the catalysts of 41%, 27%, and 17% of the reactions in Reactome, BiGG and EHMN, respectively, are not linked to an EC number. In both cases this may be because IUBMB has not yet assigned an EC number to the enzyme. For more than half of these reactions not linked to an EC number in BiGG, the catalyst facilitates a transport reaction. In this case the Transport Classification (TC) system [37] of the IUBMB might provide a more appropriate descriptor. In EHMN and Reactome this is even 73% and 70% of the cases, respectively. A number of EC numbers are missing because the database curators did not enter them into the database.

#### Miscellaneous

Next to the reasons outlined above, we also identified a number of more subtle and less frequent explanations for the limited overlap. An example at the metabolite level is that BiGG uses D-glucose in its reactions instead of specifying whether it is α-D-glucose or β-D-glucose, while Reactome only uses α-D-glucose. The other databases use all three variations. On the other hand, BiGG does not use generic metabolites like 'an alcohol' (KEGG Compound ID: C00069) or 'an L-amino acid' (KEGG Compound ID: C00151) in contrast to HumanCyc, KEGG and EHMN. Furthermore, BiGG and HumanCyc explicitly state that their reactions are charge and mass balanced. The chemical formula and charge of the metabolites were based on their ionization state at a pH level of 7.2 and 7.3, respectively, while the other three databases use the neutral form of the metabolites. This partly explains the observed increase in consensus when we did not take into account H^+^. By using the KEGG Compound ID as the prime identifier for matching metabolites, we reduce the impact of a difference in protonation state as in general the distinction between the base and the acid form of a metabolite is not made in KEGG Compound in contrast to, *e.g.*, ChEBI. We also compared the databases while allowing for an inexact match of the chemical formula with respect to the number of H atoms, to account for the variation in protonation state between the databases. This hardly affected our results (data not shown).

## Discussion

Our comparison revealed that there is only a small core of the metabolic network on which all five databases agree. Especially on reaction level the overlap is surprisingly low, only 199 reactions could be found in all five databases. Our analysis shows that the small overlap between the databases is partly explained by conceptual differences like a difference in coverage of the metabolic network. One clear example is the large set of transport reactions and reactions in lipid metabolism in EHMN, which account for 23% of the unique reactions.

Our decision to compare five pathway databases, also limits the consensus: the more databases one includes in the comparison, the lower the consensus is likely to be. We indeed observe a substantial increase in overlap when we compare pairs of databases (Additional file 4) instead of five. However, also in this case with a median consensus of around 15%, the agreement on reaction level is still relatively low. Two main factors can strongly bias the size of the consensus detected. Firstly, the consensus is constrained by differences in database size. This partly explains, for example, the consensus of only 11% when comparing a large database such as EHMN and a small database such as Reactome. Secondly, the consensus is positively influenced by the fact that databases are not constructed independently from each other. For example, EHMN used KEGG as a starting point for its reconstruction [9], which explains the higher consensus of 28%. However, even if we would restrict our comparison to three pathway databases, BiGG, EHMN, and KEGG, that are most interdependent [7,9], the consensus on reaction level is still only 14%, when not considering the transport reactions from BiGG and EHMN.

Despite the observed lack of overlap, the GO enrichment analysis of the consensus and majority genes (Additional file 2) does provide us with evidence that there is a core of metabolic processes the databases agree on. Examples of such processes are nucleotide metabolism and carbohydrate metabolism, which is also reflected on reaction level (Additional file 3). The comparison of the core metabolic processes indeed showed a considerable increase of the majority score at the gene level and to a lesser extent at reaction level. However, the consensus on reaction level remains low even for this more limited set.

Especially on reaction level the comparison is clouded by several conceptual differences and technical difficulties. The main technical challenge is to establish the identity of metabolites between databases. This was also observed to be one of the main problems for the experts involved in the construction of the consensus of two *in silico *metabolic network reconstructions of *S. cerevisiae *[24]. Matching metabolites by name is not an ideal solution, as many, possibly ambiguous, synonyms and spelling variants exist for the same metabolite [36]. Matching metabolites using metabolite identifiers is, in our comparison, restricted by the relatively large number of metabolites that had not been linked any of the four metabolite databases (KEGG, ChEBI, PubChem Compound, and CAS). One reason for the lack of metabolite identifiers is that the metabolite databases themselves are also work in progress. Metabolites that exist in a large number of structural variations such as, for example, lipids may not have been described yet in full detail in the metabolite databases. This was indeed observed for EHMN, where a large set of the unique metabolites without an identifier is involved in lipid metabolism. On the other hand, part of the metabolites of the pathway databases may not be described in any of the four metabolite databases we considered, because they, for example, do not meet the criteria to be included, such as proteins encoded by the genome found in Reactome. Furthermore, all pathway databases have a preference for one of the metabolite databases for which they curate the link. For example, BiGG mainly derived its identifiers from KEGG Compound. Similarly, for Reactome only ChEBI IDs have been manually curated. Due to this, metabolites may not link out to a metabolite database if the metabolite does not exist in the preferred reference database.

It will require a considerable manual effort to correctly assign metabolite identifiers to each metabolite and establish the correspondence of metabolites between databases. An initiative that could aid in solving some of these problems is ChemSpider [38], which integrates a wide variety of metabolite databases. The use of database-independent structural representations such as SMILES and InChI strings has also been recommended [24]. In our case, three databases (EHMN, HumanCyc and KEGG) provide InChI strings for 77%, 58%, and 75% of their metabolites, respectively. The consensus is, however, only 66 of the 3475 InChI strings in total. The low consensus when matching on InChI string can partly be explained by a difference in the amount of detail with which the structure of metabolites has been described and a difference in protonation state.

The question remains to what extent the reaction consensus would increase, even if all metabolites were properly described. As illustrated by our comparison of the TCA cycle also conceptual differences play an important role in explaining the lack of overlap. A similar conclusion can be drawn from a comparison of the two yeast metabolic networks that were used in building a consensus network [24]. Even after the identity of the metabolites between the two reconstructions had been established manually, the consensus on reaction level was still only 36%. In a recent comparison of two metabolic networks of *A. thaliana *[39] only 33% of the total number of reactions could be matched unambiguously. Furthermore, it is important to keep in mind that even if we would find unambiguous descriptions for each metabolite this does not guarantee a match. Firstly, the databases, or more specifically their metabolites, are partly complementary. EHMN, for example, explicitly focused on expanding lipid metabolism in comparison to KEGG [9]. Secondly, many of the reactants without a metabolite identifier are part of reactions that are peripheral to metabolism proper, such as precursor and degradation products of BiGG and proteins in Reactome, and are therefore unlikely to have a match in all five databases.

An example of a conceptual difference is the variation in the number of intermediate steps used to describe a specific metabolic conversion. This could be because of different database-specific criteria for when the intermediate steps of a conversion should be described or not. A second example is the use of generic metabolites (*e.g.*, alcohol) in reactions, as HumanCyc does. This may be done to model the broad substrate specificity of the enzyme or to indicate that the exact substrate specificity is unknown. Other databases, for example BiGG, focus more on indicating the specific metabolite, *e.g.*, ethanol instead of alcohol. This difference may be amplified by the number of specific instances given. Also more subtle conceptual differences play a role, like a different protonation state (neutral versus charged), the detail in which the structure of a metabolite is described (*e.g.*, D-Glucose versus α-D-Glucose) or whether the metabolite is described as enzyme bound or not (*e.g.*, lipoamide-E versus lipoamide). Finally, our GO enrichment analysis showed that the scope of the metabolic networks described by the five databases differs. The set of genes that are only found in at most two databases is, compared to the genes found in the majority of the databases, enriched for terms related to protein metabolic processes, like protein phosphorylation, proteolysis, and RNA metabolism (Additional file 2). EHMN and HumanCyc, for example, both include a generic reaction describing the phosphorylation of a protein, which is connected to a large set of 250 and 304 kinases, respectively. Differences in the metabolic processes covered by the databases also explain to some extent the differences in size of the databases.

The differences mentioned above not only make it difficult to determine the consensus between databases, but also to distinguish between conflicting and complementary content. This is especially so if one also keeps in mind that all five databases are work in progress. For example, a difference in the coverage of the metabolic network could be caused by a fundamental disagreement on whether certain processes are part of the human metabolic network. It could also be that they just did not include these processes yet and then this could be seen as complementary information. Similarly, for 45% of the consensus reactions the databases do not fully agree on the genes coding for the catalyst (Additional file 3), which may point to either complementary or conflicting information. Another example is the difference in number of steps, which can in most cases be explained by a difference in the level of detail of the description. It could, however, also reflect disagreement on the number of intermediate steps required for a particular conversion.

The low level of consensus provides compelling evidence that additional curation and the integration of the content of the five pathway databases in a single human metabolic network is desired and would improve the description of human metabolism. However, given the results of our comparison and all difficulties outlined above, what would be the way forward towards an integrated network? The consensus consists of only 199 reactions, even less when also considering the connected genes and EC numbers, and is therefore not of direct practical use. Another option is to take the union of the reactions contained in the individual databases. This is the approach taken by, for example, ConsensusPathDB [40] for integrating functional interactions, including metabolic reactions. Besides being restricted by the same conceptual and technical issues that we described, combining the content of the databases is not the definite answer. It will not solve disagreements between databases regarding, for example, the gene product catalyzing a reaction or whether a reaction can take place in human or not. Conflicting information would end up in the union and ultimately requires manual curation or at least annotation of such conflicts. Reasons for disagreement are manifold and database-dependent. Some databases, for example HumanCyc, prefer to err on the side of false positives to bring potential pathways to the attention of the community [11]. In BiGG, some reactions without evidence were included because they improved the performance of the *in silico *model. A different interpretation of the literature used in the construction of the network also causes disagreements [24]. Moreover, some parts of the metabolic network are still subject of debate and the current literature reflects these different opinions. The union will for a large part consist of data that is only supported by one of the databases.

A third option is to only include reactions on which the majority of the databases agree. This gives a higher level of confidence and in our case also a considerably larger set of 1004 reactions instead of the 199 reactions in the consensus. However, caution is warranted as for instance the databases are not strictly independent as illustrated by our pairwise comparison of KEGG and EHMN, for example. Erroneous data may, therefore, be propagated in multiple databases. Our case study of the TCA cycle also illustrates the problems of the majority vote strategy (Additional file 12). If we retain all entities the majority agrees on, 40% of the reactions are included. However, the genes MDH1 and ACO1 encoding for cytosolic proteins are also part of the majority as is the conversion of citrate to oxaloacetate (EC 2.3.3.8), which is also cytosolic. Moreover, there is no majority for any of the EC numbers proposed by one of the databases for the conversion of 2-oxoglutarate to succinyl-CoA. Also conceptual differences can be observed as, for example, we are left with two routes for both the conversion of citrate to isocitrate. Furthermore, reactions that are not part of the majority, but only found in one or two databases are not necessarily incorrect, but could be valuable complementary information. For example, KEGG gives a more detailed description of the conversion of 2-oxoglutarate to succinyl-CoA.

If the conceptual differences and technical issues we identified would be resolved the overlap will increase. It will, however, remain very difficult to (automatically) discern useful complementary information from conflicting information. In this respect, a more widespread use of evidence codes indicating the type of evidence supporting the data would enable to make a distinction between high and low confidence data. However, extensive annotation of evidence is currently only provided by BiGG and HumanCyc.

Significant manual intervention will be needed to reach the ultimate goal of a single human metabolic network. A promising model is a community-based approach, such as WikiPathways [10] or an annotation jamboree as advocated by Mo and Palsson [1]. A wiki-based approach allows the community to curate existing pathways and add new ones. Annotation jamborees are organized around domain experts and facilitate the reconciliation and refinement of metabolic pathway databases. They have already been carried out successfully for various organisms [23-25]. The results of our comparison could be used as a stepping stone for such an effort as it is crucial to understand the underlying causes of the differences to be able to resolve them. For integration purposes, we also provide an automatically derived overview of all reactions in which matching reactions are aligned, along with their associated genes, EC number and pathways (Additional file 13). The overviews of the comparison on gene, EC number and reaction level can be also found online http://www.molgenis.org/humanpathwaydb. Here, results of the comparison can be queried, sorted, and exported in a number of ways. The web application was generated using the MOLGENIS toolkit [41] and next to the graphical user interface also provides several scriptable interfaces, *e.g.*, an R interface. Using, for example, the majority reactions as a starting point for curation these overviews could aid experts on the human metabolic network to consolidate the differences between the networks and arrive at a unified model of human metabolism.

## Conclusions

An accurate and complete reconstruction of the human metabolic network is of utmost importance for its successful application in the life sciences. Our results will help curators to even further improve the metabolic network as described in the individual databases. Furthermore, as our analysis shows, each of the five pathway databases discussed in this paper provides us with a valuable piece of the puzzle. Combining the expert knowledge put into these five reconstructions and the evidence provided will improve our understanding of the human metabolic network. However, we explicitly identified many issues that prohibit the (automatic) integration of the metabolic networks. Not only the unambiguous identification of metabolites is required but the conceptual differences need to be addressed as well. Considerable manual intervention and a broad community effort are needed to reach the ultimate goal of a consolidated and biologically accurate model of human metabolism. Community efforts, such as BioPAX [42] and SBGN [43], which standardize the representation of the pathway databases, could also aid the integration of the databases. Our detailed comparison of five metabolic networks and the identification of the conceptual differences between the databases provide a stepping stone for their integration. The construction of such an integrated network will, however, require considerable time and effort. It would therefore be advisable that users keep in mind, for now, the large differences found and carefully weigh their decision when choosing a particular database or if possible apply their analyses to multiple networks to ensure the robustness of the results.

## Methods

### Data retrieval

For each of the five pathway databases, we retrieved all metabolic reactions with their corresponding gene(s), EC numbers, and pathway(s). All files mentioned below were downloaded in May, 2011.

For the metabolites we retrieved the following, most frequently provided, types of identifiers, if available in the specific pathway database (Additional file 8): KEGG Compound http://www.genome.jp/kegg/compound/, KEGG Glycan http://www.genome.jp/kegg/glycan/, ChEBI http://www.ebi.ac.uk/chebi/, PubChem http://pubchem.ncbi.nlm.nih.gov/, and CAS Registry Numbers (proprietary, assigned by the CAS registry, http://www.cas.org/). There are two types of PubChem IDs, Substance and Compound. Substance IDs are specific for the depositor of the metabolite. Compound IDs unite the different Substance IDs for the same metabolite. We used the CID-SID file ftp://ftp.ncbi.nih.gov/pubchem/Compound/Extras/CID-SID.gz to convert PubChem Substance IDs to PubChem Compound IDs.

For genes we retrieved the Entrez Gene ID, which is the only type of gene identifier the databases have in common (Additional file 8).

#### Syntactically incorrect and out-of-date identifiers

We manually corrected seven syntactically incorrect KEGG Compound IDs and 50 KEGG Glycan IDs in BiGG. We did the same for seven CAS IDs in BiGG and one in HumanCyc. For the KEGG Compound, KEGG Glycan, ChEBI and PubChem Compound IDs we checked if the IDs were up-to-date (Additional file 1). For the KEGG IDs we used the 'compound', 'glycan' and 'merged_compound.lst' file. For ChEBI we used its SQL database ftp://ftp.ebi.ac.uk/pub/databases/chebi/generic_dumps/ and for PubChem the Batch Entrez from the NCBI website http://www.ncbi.nlm.nih.gov/sites/batchentrez. We also checked and, when necessary, updated Entrez Gene IDs (Additional file 1) using the 'gene_info' and 'gene_history' files from the FTP site ftp://ftp.ncbi.nih.gov/gene/DATA/ of Entrez Gene. Finally, also the EC numbers were updated using the 'enzyme.dat' file downloaded from Expasy ftp://ftp.expasy.org/databases/enzyme/. If an out-of-date metabolite ID, Entrez Gene ID or EC number had been transferred, we replaced it with the new one, and otherwise the ID or EC number was not taken into account in the comparison.

#### BiGG

We downloaded the flat files containing reactions and metabolites from http://bigg.ucsd.edu/ [44]. We removed the 406 exchange reactions, indicated by the prefix 'EX-', added to BiGG for simulation purposes. Gene information was extracted from the SBML file. We ignored the suffix that was added to the Entrez Gene IDs to discern transcript variants. We removed 38 reaction duplicates that only differed in their tissue annotation. We raised the total percentage of metabolites with an identifier from 53% to 66% by parsing the HTML files of the metabolite pages available from the BiGG website.

#### EHMN

We downloaded from http://www.ehmn.bioinformatics.ed.ac.uk/ the EHMN Excel file containing sheets in which the reactions are linked to: (i) pathway(s) (ii) genes, represented by an Entrez Gene ID, (iii) EC number(s). A separate file was provided to us by the curators of this database containing information about the metabolites including the five types of identifiers mentioned above.

#### HumanCyc

We used Pathway Tools [45] to export the content of HumanCyc into flat files. These were combined using the internal Pathway Tools identifiers. We excluded two signaling pathways, *i.e.*, the 'BMP Signalling Pathway' and the 'MAP kinase cascade'. HumanCyc uses classes as substrates in some reactions (*e.g.*, an amino acid, an alcohol) as a way of catering for enzymes with broad substrate specificity or enzymes for which the exact substrate specificity is unknown. For the metabolite comparison we retrieved the instances provided for each metabolite class. There are 563 metabolite classes that do not have instances, of which 192 have a metabolite identifier, *e.g.*, a KEGG Compound ID. To retrieve the identifiers for these metabolite classes we used the Lisp API as they were not available in the exported flat files. Finally, the Entrez Gene ID is missing for 605 genes. If provided, the Ensembl Gene ID was mapped to an Entrez Gene ID, if available, via Ensembl BioMart (181 genes). If both gene identifiers were absent the UniProt ID was mapped to an Entrez Gene ID via the UniProt ID Mapping service (101 genes). After mapping an Entrez Gene ID was still missing for 323 genes and these were therefore not included in the comparison. For 82% of this set all three IDs mentioned are missing.

#### KEGG

We selected all human pathways from the metabolism category. For each pathway, we downloaded from the KEGG FTP site the human-specific KGML file, from which we retrieved the genes, and the KGML file containing the reference pathway linked to the EC numbers. Entries in both files are numbered, which we used to link genes to their associated EC numbers. In both files, the catalyzed reaction can be found. A single entry can contain more than one reaction, gene, and/or EC number. In that case, we assigned all genes and EC numbers contained in the entry to each reaction. Note that we cannot retrieve spontaneous reactions and reactions for which the human gene encoding the catalyst is unknown. Since KGML files only contain the main metabolites of a reaction, we retrieved the complete reaction from the flat 'reaction' file available on the FTP site. We used the 'H.sapiens.ent' file to get the Ensembl Gene IDs, and the 'compound' and 'glycan' files to extract ChEBI, PubChem Substance, and CAS IDs for metabolites.

#### Reactome

We used the dump file of the MySQL database to retrieve data from Reactome. From the top-level pathways on the front page of the Reactome website, we selected the ten pathways focused on (normal) metabolic processes, excluding, *e.g.*, signaling and disease-related pathways (see Additional file 14 for a complete list). We retrieved all reactions assigned to the selected metabolic pathways. EC numbers were obtained from the table that links catalyst activity to a GO term. Reactome contains reactions operating on sets of metabolites. We retrieved the instances of these sets from the MySQL database dump. Following the description from the Reactome Curator Guide http://wiki.reactome.org/index.php/Reactome_Curator_Guide we instantiated the reactions by taking the first member of the set at the left hand side and the first member of the set at the right hand side, and so on. In five cases this was not possible and we, therefore, did not instantiate the sets in these five reactions. Two examples are shown in Additional file 15. Reactome's black box events represent reactions for which the molecular details are not specified or unknown. We excluded a black box event if the input or output of the reaction was unknown.

#### TCA Cycle

Two EC numbers only mentioned in the comment field of the SBML file of BiGG were also taken into account. We left out the transport reactions that EHMN included in this pathway as KEGG does not contain any transport reactions in its metabolic network.

### Pathway database comparison

We compared five metabolic pathway databases at different levels: genes, EC numbers, metabolites, reactions, and relations between these components. Below, we describe in detail how we compared each of these components.

#### Genes

For the primary comparison at gene level we used Entrez Gene IDs, since it is the only gene identifier common to all five databases. BiGG, HumanCyc, and Reactome provide syntactic mechanisms for defining protein complexes, while EHMN and KEGG do not. Therefore, we did not make a distinction in the comparison between genes encoding a component of a catalyst or genes that encode a single protein catalyst.

#### EC numbers

A fully specified EC number consists of four numbers separated by a period [27]. The first three numbers indicate increasingly narrower classes and the fourth number is the serial number of the enzyme in its subclass. The databases combined contain 83 partial EC numbers, such as 1.1.1.-, which were excluded from the comparison, since they are semantically ambiguous [46].

#### Metabolites

Establishing identity between metabolites is a challenging task. For the comparison we, in general, used the KEGG Compound ID, which is in each database the most frequently provided metabolite identifier. However, KEGG Compound IDs are not available for each metabolite (Additional file 9). If the KEGG Compound ID was not provided, metabolites were matched on any of the other metabolite identifiers (KEGG Glycan, ChEBI, PubChem Compound or CAS) or metabolite name, in the latter case we also required an exact match of the chemical formula. Matching was case-insensitive and spaces and punctuation in the metabolite names were ignored. Furthermore, we computed the transitive closure of the metabolite matches. This means that if for a particular metabolite there was a match between database A and B, *e.g.*, on CAS ID, and between database B and C on, *e.g*., ChEBI ID then the metabolite was considered to match between database A and C as well. Instances of metabolite classes in HumanCyc and members of sets in Reactome were included in the comparison at metabolite level. To make the comparison as accurate as possible we did not match more generic metabolites, like alcohol or glucose, with more specific metabolites, like ethanol or α-D-glucose.

#### Reactions

We considered reactions to be the same if all substrates and products matched (see above). The direction of a reaction was not taken into account in the comparison. The same reaction written in two directions was counted as one reaction. Compartment(s) were not considered as well. We again took the transitive closure for the reaction matches (see above).

#### TCA cycle

In our detailed comparison of the TCA cycle, the following three pairs of metabolites were considered to match despite not having the same KEGG Compound ID: s-succinyldihydrolipoamide-E and s-succinyldihydrolipoamide; lipoamide-E and lipoamide; dihydrolipoamide-E and dihydrolipoamide. The only difference between these pairs is that one is the enzyme bound form of the metabolite, *e.g.*, lipoamide-E, and the other is indicated as being unbound, *e.g.*, lipoamide. The reactions were compared while not taking into account H^+^. In contrast to the comparison of the entire networks we removed neither the obsolete Entrez Gene IDs nor the gene for which the Entrez Gene was not available at all.

### Gene ontology analysis

Differences in GO biological process annotation between two lists of genes were assessed with the FatiGO functional enrichment module of the Babelomics suite (version 4.2, http://babelomics.bioinfo.cipf.es/) FatiGO uses the Fisher's exact test for 2 × 2 contingency tables to check for significant over-representation of GO biological process terms (levels 3-9) in one of the sets with respect to the other one. We used the default settings except that we set the filter for the minimum and maximum number of annotated IDs per term to 1 and 10000, respectively. GO terms were considered to be significantly over-represented if the p-values, adjusted for multiple testing by using Benjamini and Hochberg's method, were <0.01.

### Grouping pathways into categories

For the comparison of the core metabolic processes, we manually assigned the pathways of each database to one of the following nine categories using the division of KEGG as a guideline: amino acid metabolism, carbohydrate metabolism, energy metabolism, glycan biosynthesis and metabolism, lipid metabolism, metabolism of cofactors and vitamins, metabolism of secondary metabolites, nucleotide metabolism, and xenobiotics biodegradation and metabolism (Additional file 7). Pathways that did not fit in any of these nine KEGG categories were assigned to the category 'Miscellaneous'. Transport reactions of BiGG, EHMN, HumanCyc and Reactome were assigned to a separate category. A reaction was considered a transport reaction if not all metabolites were localized in the same compartment. Most transport reactions in BiGG and Reactome were originally already assigned to separate transport pathways by the databases themselves. Reactions that were not assigned to any pathway could not be assigned to any category. Note that reactions, EC numbers and genes may be found in multiple pathways and consequently may be part of multiple categories.

## Abbreviations

BiGG: Biochemically, genetically, and genomically structured; BioPAX: Biological Pathway Exchange; CAS: Chemical Abstracts Service; ChEBI: Chemical Entities of Biological Interest; EC: Enzyme Commission; EHMN: Edinburgh Human Metabolic Network; GO: Gene Ontology; InChI: International Chemical Identifier; KEGG: Kyoto Encyclopedia of Genes and Genomes; KGML: KEGG Markup Language; NC-IUBMB: Nomenclature Committee of the International Union of Biochemistry and Molecular Biology; SBGN: Systems Biology Graphical Notation; SBML: Systems Biology Markup Language; SMILES: Simplified Molecular Input Line Entry System; TCA: tricarboxylic acid.

## Authors' contributions

MDS implemented the in-house database, performed the pathway database comparison and the TCA cycle case study, interpreted the results, and drafted the manuscript. SMH interpreted the results from the TCA cycle case study and critically reviewed the manuscript. GAJ provided expert advice for the grouping of pathways into categories and critically reviewed the manuscript. AHCK and PDM conceived the study, interpreted the results, and helped draft the manuscript. All authors read and approved the final manuscript.

## Supplementary Material

Additional file 1 **Transferred and obsolete identifiers and EC numbers per database**. Number of transferred and obsolete EC numbers, gene and metabolite identifiers for each of the five pathway databases. Click here for file

Additional file 2 **Results of the FatiGO analyses**. GO biological processes enriched according to FatiGO for the following comparisons: WS1) genes in the consensus on gene level versus the union of the remaining genes, WS2) all unique genes versus the union of the remaining genes, WS3-WS7): unique genes per database (BiGG, EHMN, HumanCyc, KEGG, Reactome) versus the remaining genes contained in the union, WS8) genes contained in the majority of the databases versus the union of the remaining genes. Click here for file

Additional file 3 **Consensus reactions**. Overview of the reactions part of the consensus of all five pathway databases (when not taking into account e^-^, H^+ ^and H_2_O). For each consensus reaction the corresponding EC numbers, genes (Entrez Gene IDs), and pathways are also given for each database. Click here for file

Additional file 4 **Pairwise comparison of the five databases on gene, EC, metabolite, and reaction level**. Consensus between pairs of databases is calculated as in the main text: (|*C*_*DB1 *_∩ *C*_*DB2*_|/|*C*_*DB1 *_∪ *C*_*DB2*_|) × 100%, where *C *is the set of entities under consideration. Databases are compared on Entrez Gene IDs, EC numbers, metabolites, and reactions, which were not required to match on e^-^, H^+ ^and/or H_2_O. Click here for file

Additional file 5 **TCA cycle as represented in each of the five metabolic pathway databases**. Adapted version of Figure 2 in the main text for each of the metabolic pathway databases separately. Reactions occurring in the TCA cycle for the selected database are highlighted. Metabolites are represented by rectangles, genes by rounded rectangles, and EC numbers by parallelograms. Color indicates how many of the five databases include a specific entity. Color of an arrow indicates the number of databases that agree upon an entire reaction, *i.e.*, all its metabolites (except H^+ ^which was matched separately). 'x' denotes a missing EC number. Click here for file

Additional file 6 **TCA cycle as represented in each of the five metabolic pathway databases**. Breakdown of the TCA cycle per database. WS1) Overview of all reactions, plus corresponding EC numbers and genes. WS2) Reactions of each database; matching reactions are aligned. For reactions that are not part of the TCA cycle consensus an explanation for the differences observed is given in column B (see also section 'Analysis of differences between databases' in the main text). WS3) Metabolites of each database; matching metabolites are aligned. WS4) EC numbers of each database; matching EC numbers are aligned. WS5) Genes of each database; matching genes are aligned. In WS3-WS5 metabolites, EC numbers, and genes are matched across the entire TCA cycle. Click here for file

Additional file 7 **Grouping of pathways into categories**. Overview of the manual grouping of pathways of each database into one of eleven categories, see Materials and Methods. Click here for file

Additional file 8 **Identifier types for genes and metabolites present in each of the databases**. Click here for file

Additional file 9 **Metabolite counts per database**. For each of the five databases the percentage of metabolites without a chemical formula and the percentage of metabolites without an identifier is indicated. Furthermore, for each pathway database the percentage of metabolites linked to a particular metabolite database (KEGG Compound, KEGG Glycan, ChEBI, PubChem Compound, and CAS) is indicated. We also included the instances of metabolite classes for HumanCyc and members of sets for Reactome, see Materials and Methods. Click here for file

Additional file 10 **Names of metabolites without a match in any of the four other databases and without any of the five types of metabolite identifiers**. Click here for file

Additional file 11 **Metabolite counts per database for the comparison of core metabolic processes**. For the metabolites of the core metabolic processes in each of the five pathway databases the percentage of metabolites without a chemical formula and the percentage of metabolites without an identifier is indicated. Furthermore, for each pathway database the percentage of metabolites linked to a particular metabolite database (KEGG Compound, KEGG Glycan, ChEBI, PubChem Compound, and CAS) is indicated. We also included the instances of metabolite classes for HumanCyc and members of sets for Reactome, see Materials and Methods. Click here for file

Additional file 12 **TCA cycle: majority vote**. Adapted version of Figure 2 in the main text when retaining only the entities that at least three out of five databases agree on. Reactions occurring in the majority are highlighted. Metabolites are represented by rectangles, genes by rounded rectangles, and EC numbers by parallelograms. Color indicates how many of the five databases include a specific entity. Color of an arrow indicates the number of databases that agree upon an entire reaction, *i.e.*, all its metabolites (except H^+ ^which was matched separately). Click here for file

Additional file 13 **Overview of all reactions and their matches**. Overview of all reactions and their matches (when not taking into account e^-^, H^+ ^and H_2_O). Rows are colored according to the number of databases that agree on a reaction. For each reaction the corresponding EC numbers, genes (Entrez Gene IDs), and pathways are also given for each database. Click here for file

Additional file 14 **Top-level pathways from Reactome (not) considered in the comparison**. Click here for file

Additional file 15 **Instantiating reactions containing sets of metabolites**. Reactome contains reactions defined in terms of sets of metabolites. For five of such reactions the specific instantiations could not be derived automatically, this document gives two detailed examples. Click here for file
