# Supplementary material for: Critical assessment of human metabolic pathway databases: a stepping stone for future integration
Source: BMC Syst Biol. 2011 Oct 14;5:165. doi: 10.1186/1752-0509-5-165 (PMC3271347; doi:10.1186/1752-0509-5-165)
Supplement: Additional file 1 — Transferred and obsolete identifiers and EC numbers per database. Number of transferred and obsolete EC numbers, gene and metabolite identifiers for each of the five pathway databases. [file 1752-0509-5-165-S1.PDF]

Additional file 1 – Transferred and obsolete identifiers and EC numbers per database

Genes

	Number of Entrez Gene IDs	
Database	transferred[a]	obsolete
BiGG	10 (1)	5
EHMN	4 (1)	24
HumanCyc	38 (19)	5
KEGG	1 (0)	0
Reactome	10 (9)	21

	Total number of Entrez Gene IDs	
Database	before update	after update
BiGG	1496	1490
EHMN	2517	2492
HumanCyc	3233	3209
KEGG	1535	1535
Reactome	1210	1180

[a] The number of the genes that were transferred to an ID that was already present in the set of Entrez Gene IDs of the particular database is indicated between brackets.

EC numbers

	Number of EC numbers		
Database	incomplete	transferred	obsolete
BiGG	2	8	1
EHMN	43	4	1
HumanCyc	34	2	0
KEGG	34	0	0
Reactome	19	3	0

	Total number of EC numbers	
Database	before update	after update
BiGG	644	645
EHMN	936	940
HumanCyc	1212	1215
KEGG	726	726
Reactome	354	356

Incomplete EC numbers were not taken into account in the comparison because of their ambiquity.
Some EC numbers were transferred to multiple new EC numbers. In Reactome there are two cases in which the new EC number was already included in Reactome.

Metabolites

Number of	KEGG Compound			KEGG Glycan		CAS	ChEBI		PubChem Compound	
Database	incorrectly formatted	transferred	obsolete	incorrectly formatted	obsolete	incorrectly formatted	transferred	obsolete	PubChem Substance IDs that do not map to a PubChem Compound ID	obsolete
BiGG	8[b]	14	21	50	2	7	x	x	x	1
EHMN	0	28	4	0	0	0	3	0	35	0[c]
HumanCyc	0	9	0	x	x	2[b]	1	39	x	106
KEGG	0	0	0	0	0	0	0	0	259	0[c]
Reactome	0	8	1	x	x	x	0	0	12	0[c]

An 'x' indicates that the particular identifier is not available for this database.
[b] One could not be corrected and was therefore removed
[c] As the CID-SID.gz file from PubChem was used to convert the PubChem Substance IDs to PubChem Compound IDs these will naturally be up-to-date.
