# Supplementary material for: Critical assessment of human metabolic pathway databases: a stepping stone for future integration
Source: BMC Syst Biol. 2011 Oct 14;5:165. doi: 10.1186/1752-0509-5-165 (PMC3271347; doi:10.1186/1752-0509-5-165)
Supplement: Additional file 4 — Pairwise comparison of the five databases on gene, EC, metabolite, and reaction level. Consensus between pairs of databases is calculated as in the main text: (|CDB1 ∩ CDB2|/|CDB1 ∪ CDB2|) × 100%, where C is the set of entities under consideration. Databases are compared on Entrez Gene IDs, EC numbers, metabolites, and reactions, which were not required to match on e-, H+ and/or H2O. [file 1752-0509-5-165-S4.PDF]

Additional file 4 – Pairwise comparison of the five databases on gene, EC, metabolite, and reaction level

Consensus between pairs of databases is calculated as in the main text:

$$\frac{|C_{DB1} \cap C_{DB2}|}{|C_{DB1} \cup C_{DB2}|} \times 100\%,$$

where *C* is the set of entities under consideration. Databases are compared on Entrez Gene IDs, EC numbers, metabolites, and reactions, which were not required to match on e^-, H^+ and/or H_2O.

genes

	BiGG	EHMN	HumanCyc	KEGG	Reactome
BiGG	x	46.4	36.0	60.7	42.5
EHMN		x	60.4	43.2	31.8
HumanCyc			x	40.1	24.6
KEGG				x	32.2
Reactome					x

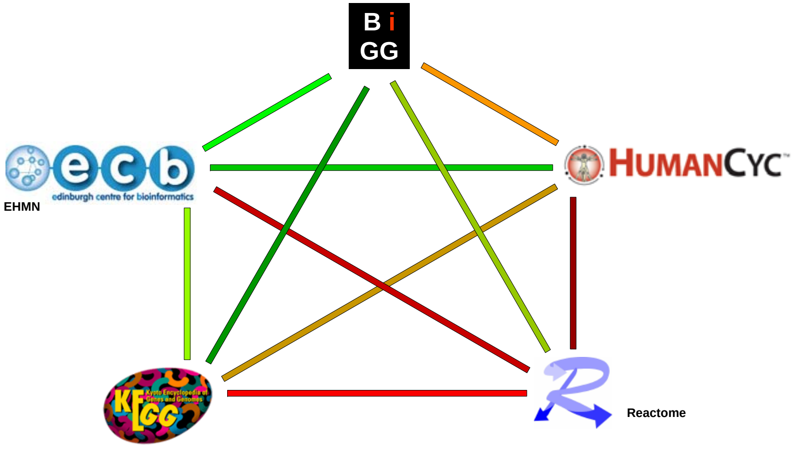

EC numbers

	BiGG	EHMN	HumanCyc	KEGG	Reactome
BiGG	x	52.4	44.4	62.8	38.3
EHMN		x	61.3	62.7	33.9
HumanCyc			x	55.8	26.7
KEGG				x	39.1
Reactome					x

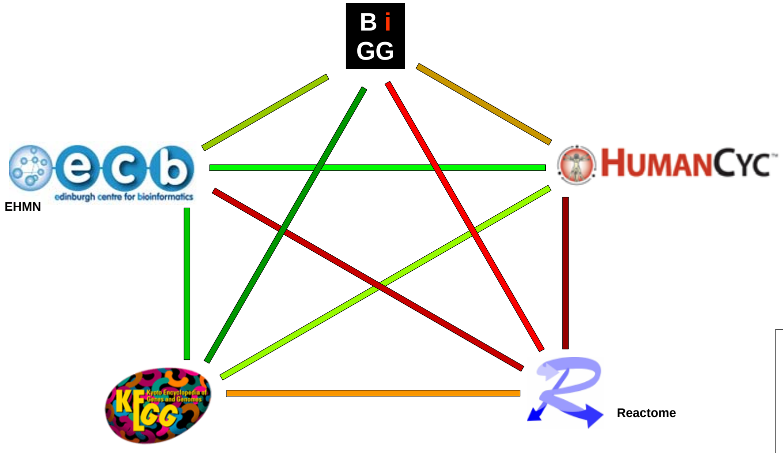

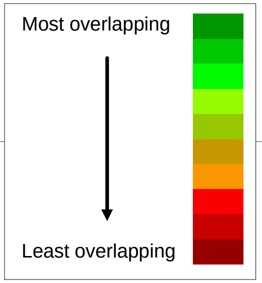

metabolites

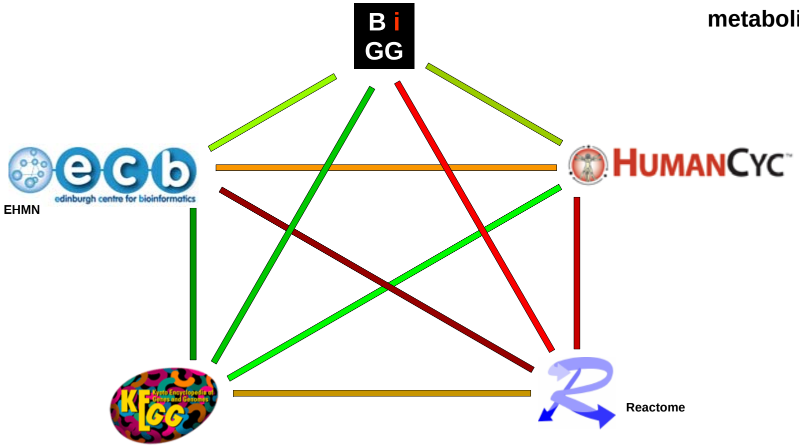

	BiGG	EHMN	HumanCyc	KEGG	Reactome
BiGG	x	27.0	26.8	36.0	23.8
EHMN		x	24.7	40.1	16.8
HumanCyc			x	28.2	21.7
KEGG				x	24.9
Reactome					x

reactions

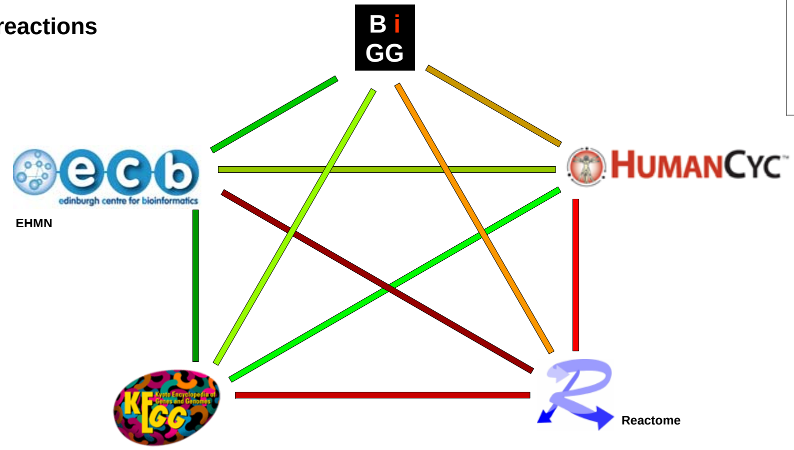

	BiGG	EHMN	HumanCyc	KEGG	Reactome
BiGG	x	22.4	14.4	17.9	14.4
EHMN		x	14.7	28.5	11.1
HumanCyc			x	20.6	13.5
KEGG				x	13.0
Reactome					x
