# Supplementary material for: Critical assessment of human metabolic pathway databases: a stepping stone for future integration
Source: BMC Syst Biol. 2011 Oct 14;5:165. doi: 10.1186/1752-0509-5-165 (PMC3271347; doi:10.1186/1752-0509-5-165)
Supplement: Additional file 5 — TCA cycle as represented in each of the five metabolic pathway databases. Adapted version of Figure 2 in the main text for each of the metabolic pathway databases separately. Reactions occurring in the TCA cycle for the selected database are highlighted. Metabolites are represented by rectangles, genes by rounded rectangles, and EC numbers by parallelograms. Color indicates how many of the five databases include a specific entity. Color of an arrow indicates the number of databases that agree upon an entire reaction, i.e., all its metabolites (except H+ which was matched separately). 'x' denotes a missing EC number. [file 1752-0509-5-165-S5.PDF]

Additional file 5 – TCA cycle as represented in each of the five metabolic pathway databases

Adapted version of Figure 2 in the main text for each of the metabolic pathway databases separately. Reactions occurring in the TCA cycle for the selected database are highlighted. Metabolites are represented by rectangles, genes by rounded rectangles, and EC numbers by parallelograms. Color indicates how many of the five databases include a specific entity. Color of an arrow indicates the number of databases that agree upon an entire reaction, *i.e.*, all its metabolites (except H^+ which was matched separately). ‘x’ denotes a missing EC number.

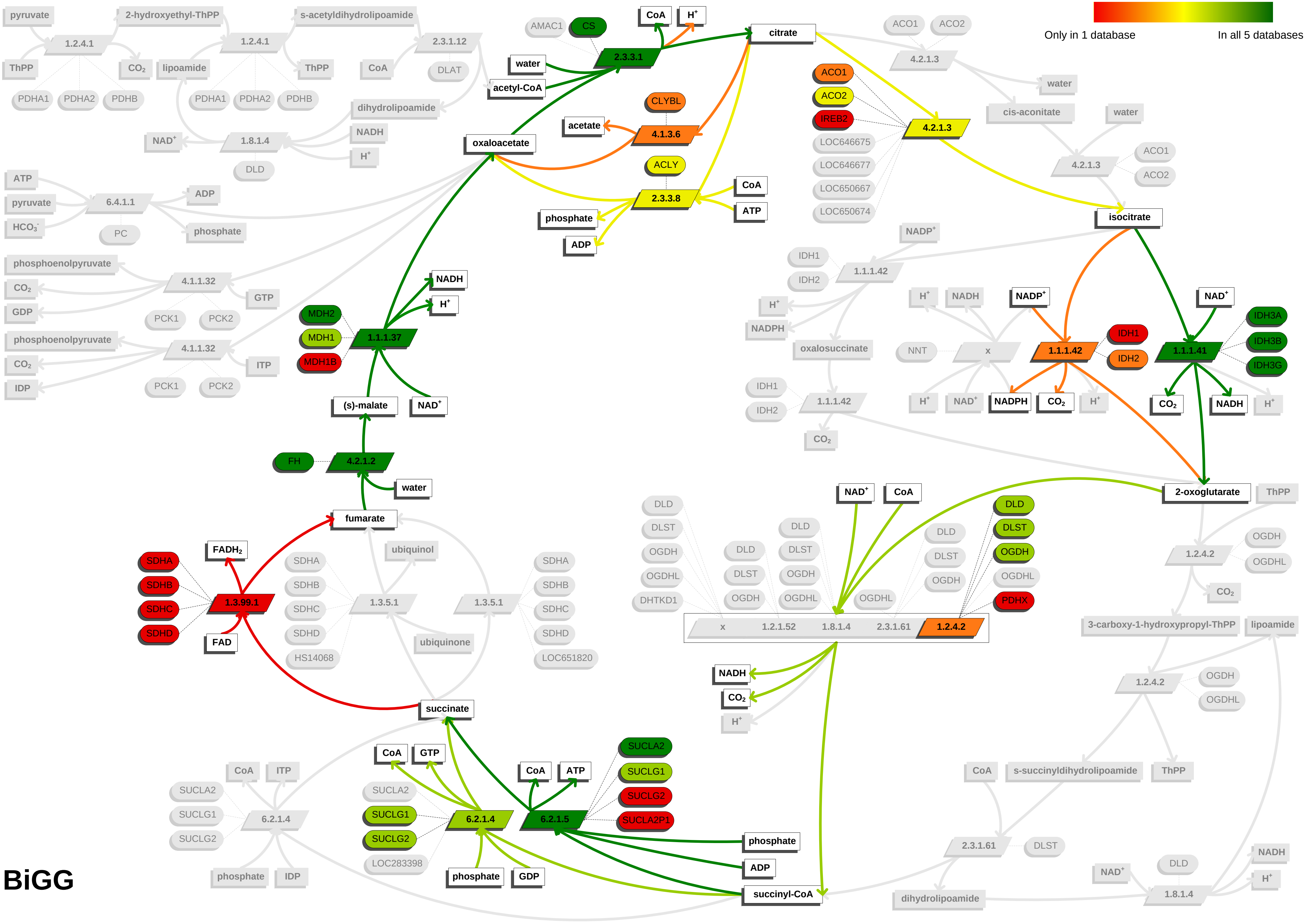

Only in 1 database
In all 5 databases
BiGG
pyruvate
2-hydroxyethyl-ThPP
s-acetyldihydrolipoamide
1.2.4.1
1.2.4.1
2.3.1.12
ThPP
CO2
lipoamide
ThPP
CoA
DLAT
PDHA1
PDHA2
PDHB
dihydrolipoamide
NAD+
1.8.1.4
NADH
H+
DLD
ATP
pyruvate
HCO3-
6.4.1.1
ADP
phosphate
PC
phosphoenolpyruvate
4.1.1.32
CO2
GTP
GDP
PCK1
PCK2
ITP
IDP
AMAC1
CS
CoA
H+
citrate
water
2.3.3.1
acetyl-CoA
oxaloacetate
acetate
CLYBL
4.1.3.6
ACLY
2.3.3.8
CoA
ATP
phosphate
ADP
NADH
H+
MDH2
MDH1
MDH1B
1.1.1.37
(s)-malate
NAD+
FH
4.2.1.2
water
fumarate
FADH2
FAD
SDHA
SDHB
SDHC
SDHD
1.3.99.1
ubiquinol
ubiquinone
1.3.5.1
HS14068
LOC651820
succinate
ACO1
ACO2
IREB2
LOC646675
LOC646677
LOC650667
LOC650674
4.2.1.3
water
cis-aconitate
isocitrate
NADP+
IDH1
IDH2
1.1.1.42
NADPH
oxalosuccinate
NNT
x
IDH3A
IDH3B
IDH3G
1.1.1.41
CO2
NADH
H+
2-oxoglutarate
ThPP
DLST
OGDH
OGDHL
DHTKD1
PDHX
1.2.4.2
1.2.1.52
2.3.1.61
3-carboxy-1-hydroxypropyl-ThPP
lipoamide
s-succinyldihydrolipoamide
CoA
SUCLA2
SUCLG1
SUCLG2
SUCLA2P1
LOC283398
6.2.1.4
6.2.1.5
GTP
phosphate
GDP
succinyl-CoA

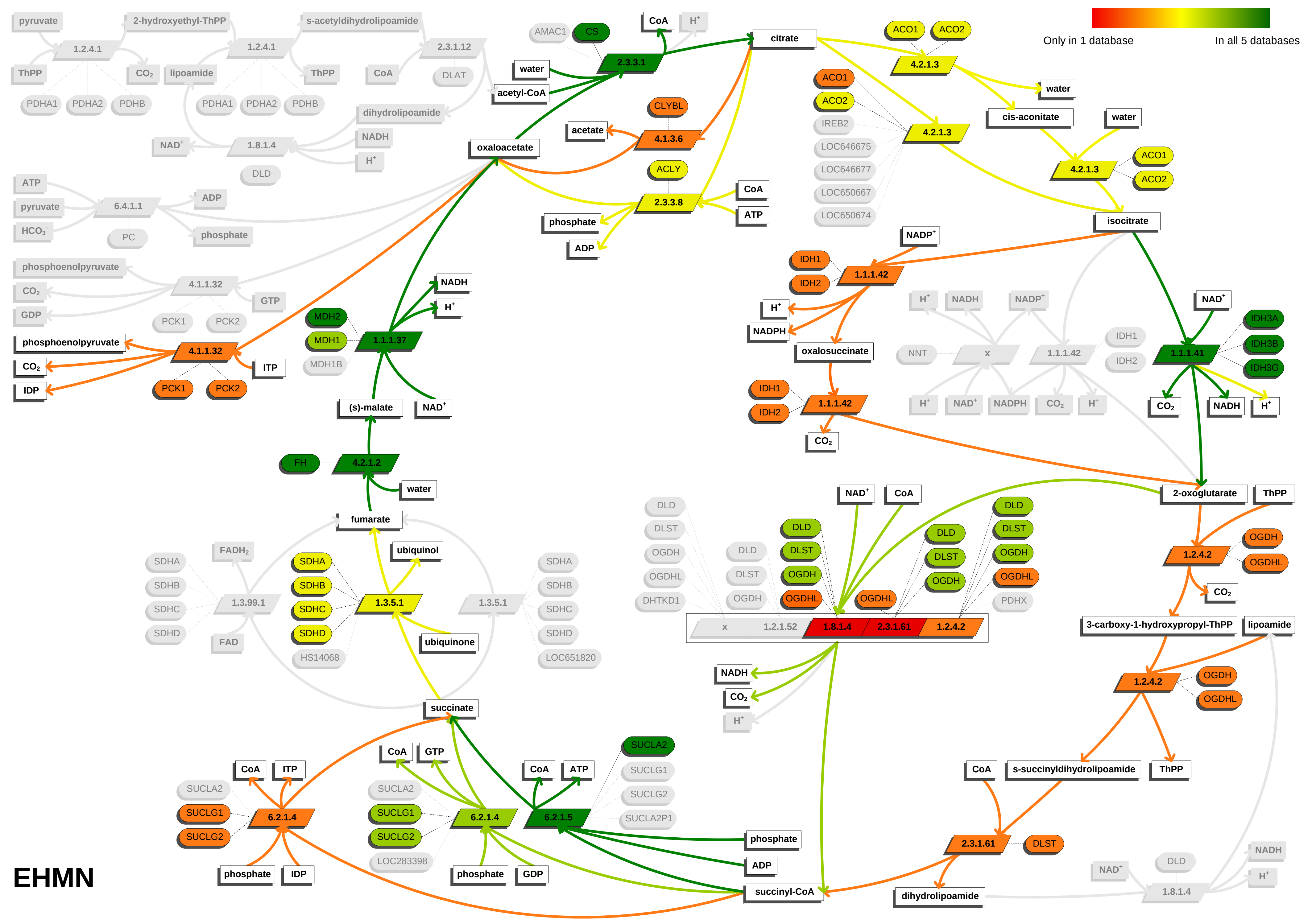

Only in 1 database
In all 5 databases
pyruvate
2-hydroxyethyl-ThPP
s-acetyldihydrolipoamide
1.2.4.1
1.2.4.1
2.3.1.12
ThPP
CO_2
lipoamide
ThPP
CoA
DLAT
PDHA1
PDHA2
PDHB
PDHA1
PDHA2
PDHB
dihydrolipoamide
NADH
NAD^+
1.8.1.4
H^+
DLD
ATP
pyruvate
HCO_3^-
6.4.1.1
ADP
PC
phosphate
phosphoenolpyruvate
CO_2
GDP
4.1.1.32
GTP
PCK1
PCK2
phosphoenolpyruvate
CO_2
IDP
4.1.1.32
ITP
PCK1
PCK2
AMAC1
CS
CoA
H^+
water
2.3.3.1
acetyl-CoA
citrate
oxaloacetate
CLYBL
acetate
4.1.3.6
ACLY
2.3.3.8
CoA
ATP
phosphate
ADP
NADH
H^+
MDH2
MDH1
1.1.1.37
MDH1B
(s)-malate
NAD^+
FH
4.2.1.2
water
fumarate
ubiquinol
FADH$_2$
SDHA
SDHB
SDHC
SDHD
1.3.99.1
FAD
SDHA
SDHB
SDHC
SDHD
HS14068
1.3.5.1
ubiquinone
1.3.5.1
SDHA
SDHB
SDHC
SDHD
LOC651820
succinate
ACO1
ACO2
4.2.1.3
water
cis-aconitate
water
ACO1
ACO2
IREB2
LOC646675
LOC646677
LOC650667
LOC650674
4.2.1.3
4.2.1.3
ACO1
ACO2
isocitrate
$NADP^+$
IDH1
IDH2
1.1.1.42
H^+
NADPH
oxalosuccinate
H^+
NADH
$NADP^+$
NNT
x
1.1.1.42
IDH1
IDH2
H^+
NAD^+
NADPH
CO_2
H^+
NAD^+
IDH3A
IDH3B
IDH3G
1.1.1.41
CO_2
NADH
H^+
IDH1
IDH2
1.1.1.42
CO_2
2-oxoglutarate
ThPP
NAD^+
CoA
DLD
DLST
OGDH
OGDHL
DHTKD1
DLD
DLST
OGDH
DLD
DLST
OGDH
OGDHL
OGDHL
DLD
DLST
OGDH
DLD
DLST
OGDH
OGDHL
PDHX
x
1.2.1.52
1.8.1.4
2.3.1.61
1.2.4.2
OGDH
OGDHL
1.2.4.2
CO_2
3-carboxy-1-hydroxypropyl-ThPP
lipoamide
1.2.4.2
OGDH
OGDHL
NADH
CO_2
H^+
CoA
s-succinyldihydrolipoamide
ThPP
2.3.1.61
DLST
dihydrolipoamide
NAD^+
DLD
1.8.1.4
NADH
H^+
CoA
ITP
SUCLA2
SUCLG1
SUCLG2
6.2.1.4
phosphate
IDP
CoA
GTP
SUCLA2
SUCLG1
SUCLG2
LOC283398
6.2.1.4
phosphate
GDP
CoA
ATP
SUCLA2
SUCLG1
SUCLG2
SUCLA2P1
6.2.1.5
phosphate
ADP
succinyl-CoA
EHMN

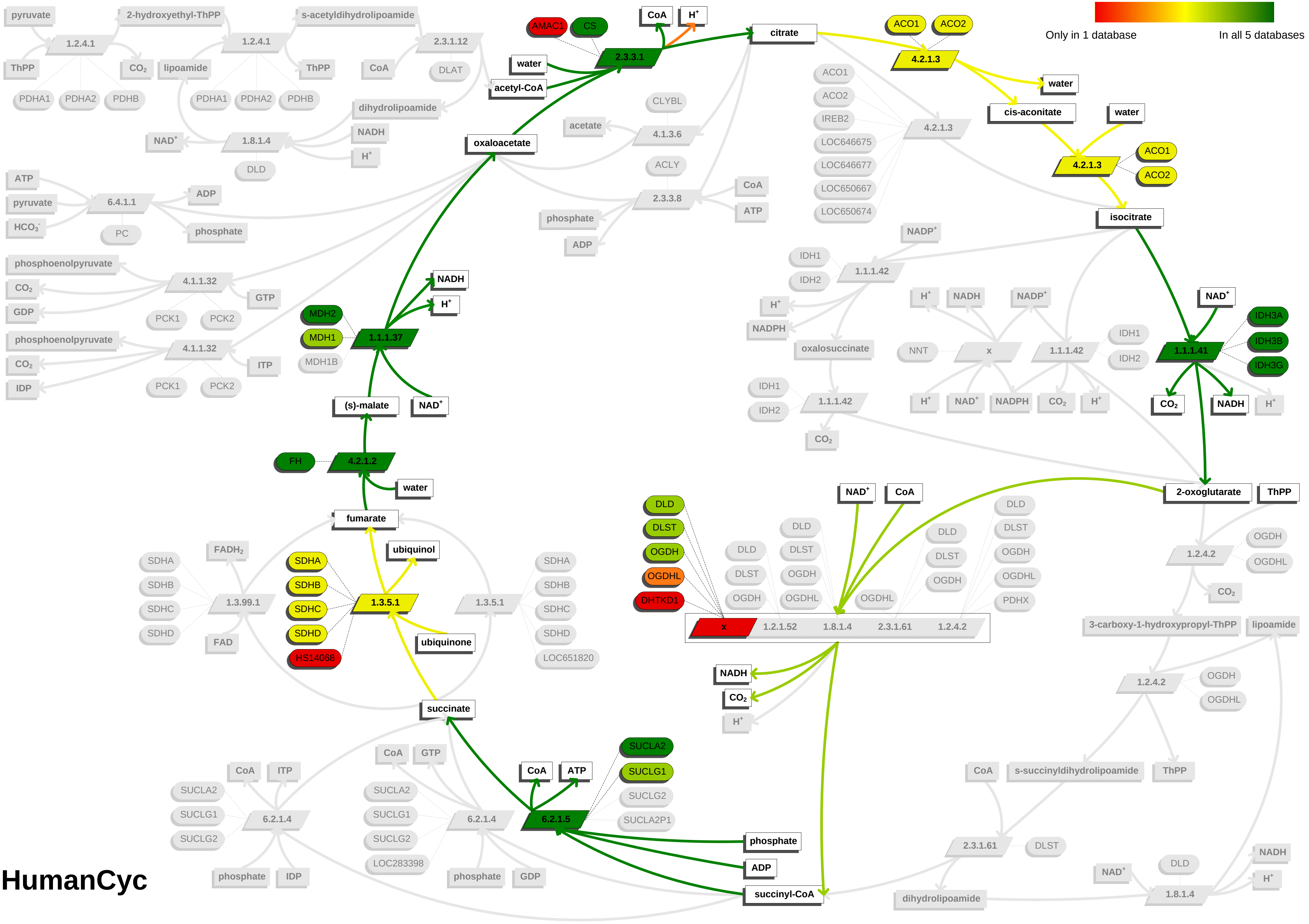

Only in 1 database
In all 5 databases
pyruvate
2-hydroxyethyl-ThPP
s-acetyldihydrolipoamide
1.2.4.1
2.3.1.12
ThPP
CO_2
lipoamide
CoA
DLAT
PDHA1
PDHA2
PDHB
dihydrolipoamide
NADH
NAD^+
1.8.1.4
H^+
DLD
ATP
6.4.1.1
ADP
HCO_3^-
PC
phosphate
phosphoenolpyruvate
4.1.1.32
GTP
GDP
PCK1
PCK2
ITP
IDP
AMAC1
CS
2.3.3.1
water
acetyl-CoA
citrate
oxaloacetate
CLYBL
acetate
4.1.3.6
ACLY
2.3.3.8
ACO1
ACO2
4.2.1.3
IREB2
LOC646675
LOC646677
LOC650667
LOC650674
cis-aconitate
isocitrate
$NADP^+$
IDH1
IDH2
1.1.1.42
NADPH
oxalosuccinate
NNT
x
1.1.1.41
IDH3A
IDH3B
IDH3G
MDH2
MDH1
MDH1B
1.1.1.37
(s)-malate
FH
4.2.1.2
fumarate
ubiquinol
ubiquinone
$FADH_2$
FAD
SDHA
SDHB
SDHC
SDHD
HS14068
1.3.99.1
1.3.5.1
LOC651820
2-oxoglutarate
DLST
OGDH
OGDHL
DHTKD1
PDHX
1.2.1.52
2.3.1.61
1.2.4.2
3-carboxy-1-hydroxypropyl-ThPP
s-succinyldihydrolipoamide
succinate
SUCLA2
SUCLG1
SUCLG2
SUCLA2P1
LOC283398
6.2.1.4
6.2.1.5
succinyl-CoA

HumanCyc

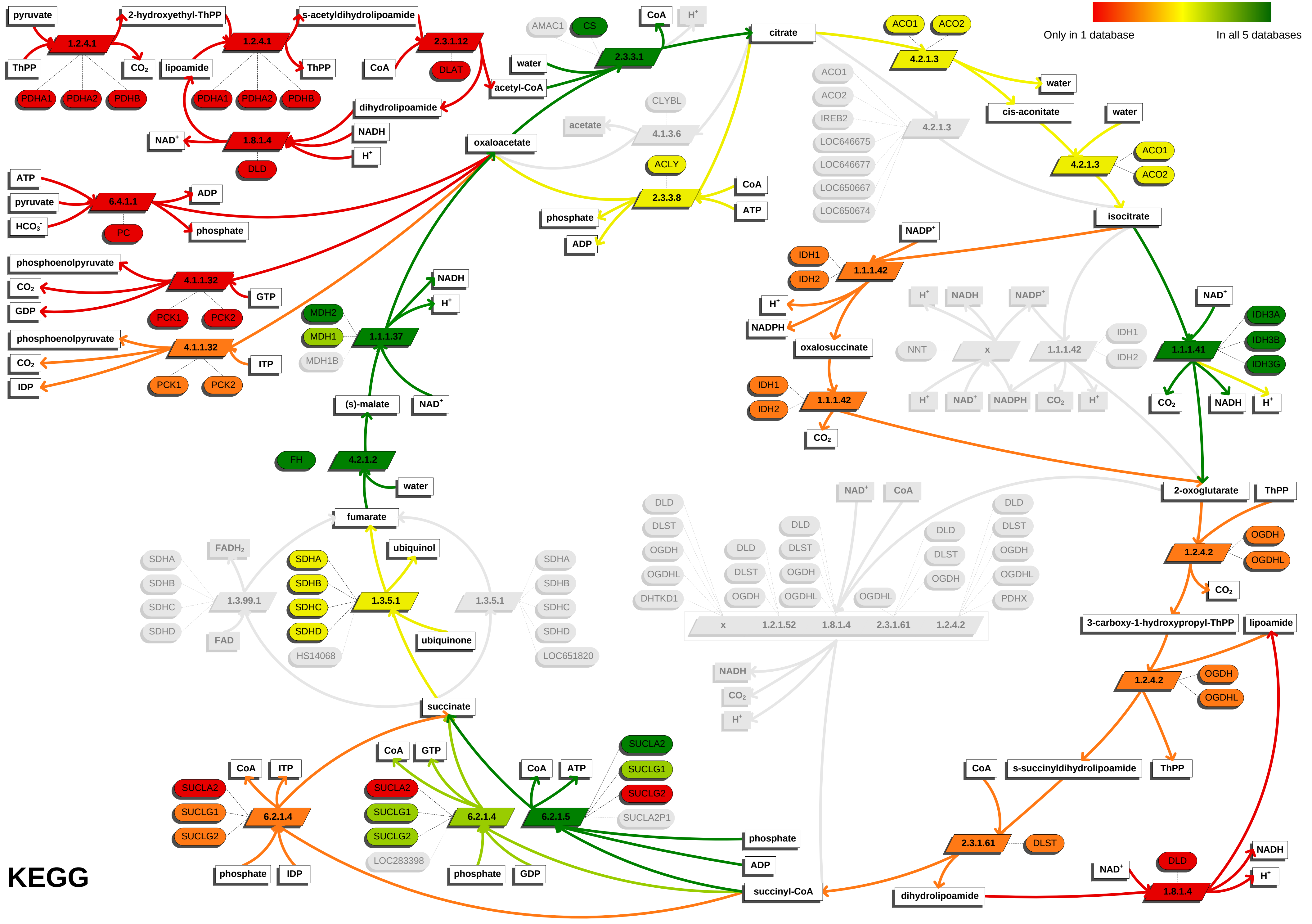

Only in 1 database
In all 5 databases
pyruvate
2-hydroxyethyl-ThPP
s-acetyldihydrolipoamide
1.2.4.1
1.2.4.1
2.3.1.12
ThPP
CO2
lipoamide
ThPP
CoA
DLAT
PDHA1
PDHA2
PDHB
PDHA1
PDHA2
PDHB
dihydrolipoamide
NADH
NAD+
1.8.1.4
H+
DLD
ATP
pyruvate
HCO3-
6.4.1.1
ADP
PC
phosphate
phosphoenolpyruvate
4.1.1.32
CO2
GTP
GDP
PCK1
PCK2
phosphoenolpyruvate
4.1.1.32
CO2
ITP
IDP
PCK1
PCK2
AMAC1
CS
CoA
H+
citrate
water
2.3.3.1
acetyl-CoA
oxaloacetate
CLYBL
acetate
4.1.3.6
ACLY
2.3.3.8
CoA
ATP
phosphate
ADP
NADH
H+
MDH2
MDH1
MDH1B
1.1.1.37
(s)-malate
NAD+
FH
4.2.1.2
water
fumarate
ubiquinol
FADH2
SDHA
SDHB
SDHC
SDHD
1.3.99.1
FAD
SDHA
SDHB
SDHC
SDHD
HS14068
1.3.5.1
ubiquinone
1.3.5.1
SDHA
SDHB
SDHC
SDHD
LOC651820
succinate
CoA
ITP
SUCLA2
SUCLG1
SUCLG2
6.2.1.4
phosphate
IDP
CoA
GTP
SUCLA2
SUCLG1
SUCLG2
LOC283398
6.2.1.4
phosphate
GDP
CoA
ATP
6.2.1.5
SUCLA2
SUCLG1
SUCLG2
SUCLA2P1
phosphate
ADP
succinyl-CoA
ACO1
ACO2
4.2.1.3
water
cis-aconitate
water
4.2.1.3
ACO1
ACO2
isocitrate
ACO1
ACO2
IREB2
LOC646675
LOC646677
LOC650667
LOC650674
4.2.1.3
NADP+
IDH1
IDH2
1.1.1.42
H+
NADPH
oxalosuccinate
IDH1
IDH2
1.1.1.42
CO2
H+
NADH
NADP+
NNT
x
1.1.1.42
IDH1
IDH2
H+
NAD+
NADPH
CO2
H+
NAD+
IDH3A
IDH3B
IDH3G
1.1.1.41
CO2
NADH
H+
2-oxoglutarate
ThPP
NAD+
CoA
DLD
DLST
OGDH
OGDHL
DHTKD1
DLD
DLST
OGDH
DLD
DLST
OGDH
OGDHL
DLD
DLST
OGDH
OGDHL
DLD
DLST
OGDH
OGDHL
PDHX
x
1.2.1.52
1.8.1.4
2.3.1.61
1.2.4.2
NADH
CO2
H+
OGDH
OGDHL
1.2.4.2
CO2
3-carboxy-1-hydroxypropyl-ThPP
lipoamide
1.2.4.2
OGDH
OGDHL
CoA
s-succinyldihydrolipoamide
ThPP
2.3.1.61
DLST
dihydrolipoamide
NAD+
DLD
1.8.1.4
NADH
H+
KEGG

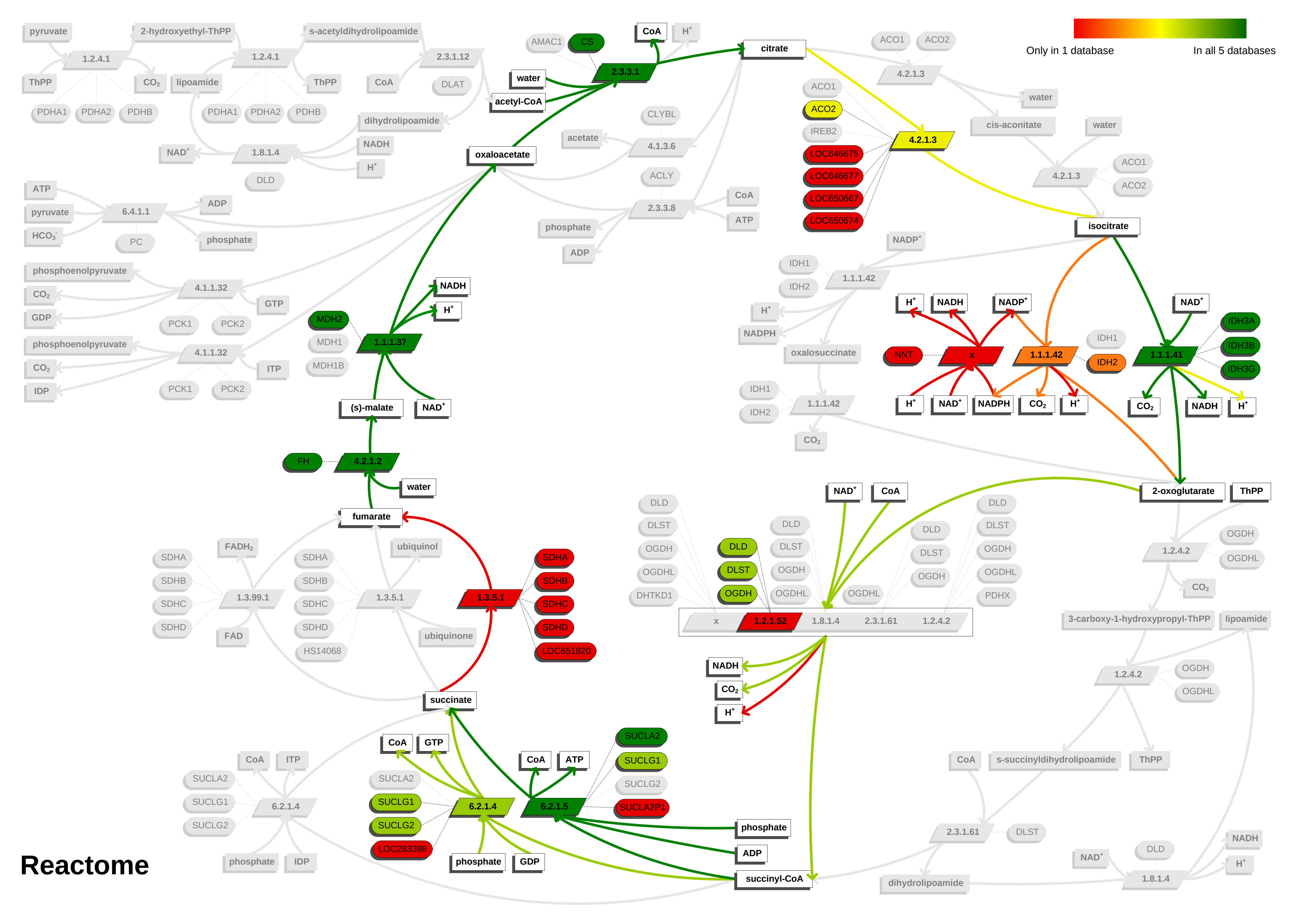
Only in 1 database
In all 5 databases
Reactome
pyruvate
2-hydroxyethyl-ThPP
s-acetyldihydrolipoamide
1.2.4.1
2.3.1.12
ThPP
CO2
lipoamide
CoA
DLAT
PDHA1
PDHA2
PDHB
dihydrolipoamide
NAD+
1.8.1.4
NADH
H+
DLD
ATP
6.4.1.1
ADP
HCO3-
PC
phosphate
phosphoenolpyruvate
4.1.1.32
GTP
GDP
PCK1
PCK2
ITP
IDP
AMAC1
CS
2.3.3.1
water
acetyl-CoA
citrate
oxaloacetate
CLYBL
acetate
4.1.3.6
ACLY
2.3.3.8
ACO1
ACO2
4.2.1.3
IREB2
LOC646675
LOC646677
LOC650667
LOC650674
cis-aconitate
isocitrate
NADP+
IDH1
IDH2
1.1.1.42
NADPH
oxalosuccinate
NNT
x
IDH3A
IDH3B
IDH3G
1.1.1.41
MDH2
MDH1
MDH1B
1.1.1.37
(s)-malate
FH
4.2.1.2
fumarate
2-oxoglutarate
DLST
OGDH
OGDHL
DHTKD1
PDHX
1.2.1.52
2.3.1.61
1.2.4.2
3-carboxy-1-hydroxypropyl-ThPP
SDHA
SDHB
SDHC
SDHD
FADH2
1.3.99.1
FAD
ubiquinol
1.3.5.1
ubiquinone
HS14068
LOC651820
succinate
SUCLA2
SUCLG1
SUCLG2
6.2.1.4
6.2.1.5
SUCLA2P1
LOC283398
succinyl-CoA
s-succinyldihydrolipoamide
