# Supplementary material for: Critical assessment of human metabolic pathway databases: a stepping stone for future integration
Source: BMC Syst Biol. 2011 Oct 14;5:165. doi: 10.1186/1752-0509-5-165 (PMC3271347; doi:10.1186/1752-0509-5-165)
Supplement: Additional file 8 — Identifier types for genes and metabolites present in each of the databases. [file 1752-0509-5-165-S8.PDF]

Additional file 8 – Identifier types for genes and metabolites present in each of the databases

	**Gene identifiers**
BiGG	Entrez Gene, HGNC (only on website and in pdf)
EHMN	Entrez Gene, Ensembl Gene, HGNC (only on website)
HumanCyc	Entrez Gene, Ensembl gene, UCSC, UniGene, Entrez, Genecards, RefSeq_NM
KEGG	KEGG Gene ID (=Entrez Gene), NCBI-GeneID (=Entrez Gene), HGNC, Ensembl, RefSeq_XM
Reactome	Entrez Gene, Ensembl Gene, UCSC, KEGG Gene ID, BioGPS, CTD

	**Metabolite identifiers**
BiGG	KEGG Compound, KEGG Glycan, PubChem Compound (in comments), PubChem Substance (in comments), CAS
EHMN	KEGG Compound, KEGG Glycan, ChEBI, PubChem Substance, CAS, InChI, SMILES, EMP ID, HMDB
HumanCyc	KEGG Compound, ChEBI, PubChem Compound, CAS, InChI, SMILES, NCI, UM-BBD-CPD, KNApSAck, NIKKAJI, Wikipedia
KEGG	KEGG Compound, KEGG Glycan, ChEBI, PubChem Substance, CAS, InChI, PDB-CCD, 3DMET, KNApSAcK, LIPIDMAPS, LIPIDBANK
Reactome	KEGG Compound, ChEBI, PubChem Substance

Note that some identifiers are only present for a few genes/metabolites.
