# Supplementary material for: Critical assessment of human metabolic pathway databases: a stepping stone for future integration
Source: BMC Syst Biol. 2011 Oct 14;5:165. doi: 10.1186/1752-0509-5-165 (PMC3271347; doi:10.1186/1752-0509-5-165)
Supplement: Additional file 9 — Metabolite counts per database. For each of the five databases the percentage of metabolites without a chemical formula and the percentage of metabolites without an identifier is indicated. Furthermore, for each pathway database the percentage of metabolites linked to a particular metabolite database (KEGG Compound, KEGG Glycan, ChEBI, PubChem Compound, and CAS) is indicated. We also included the instances of metabolite classes for HumanCyc and members of sets for Reactome, see Materials and Methods. [file 1752-0509-5-165-S9.PDF]

Additional file 9 – Metabolite counts per database

Database	total number of metabolites	% of metabolites without a chemical formula	% of metabolites without identifier	# of metabolites with identifier	% metabolites with				
					KEGG Compound	KEGG	ChEBI	PubChem Compound	CAS
BiGG	1485	0	34	984	87	15	0	3	57
EHMN	2676	10	32	1830	91	6	49	33	40
HumanCyc	1681	18	25	1258	88	0	49	77	40
KEGG	1553	10	0	1553	92	14	67	75	47
Reactome	984	44	31	682	85	0	98	54	0

We also included the instances of metabolite classes for HumanCyc and members of sets for Reactome, see Materials and Methods. The percentages of the different types of metabolite identifiers are calculated w.r.t. the number of metabolites with at least one identifier. Note that in KEGG each metabolite has at least an identifier from one of its own metabolite databases, KEGG Compound and KEGG Glycan. For 16% of the metabolites of KEGG there is no other identifier provided.
