# Supplementary material for: Critical assessment of human metabolic pathway databases: a stepping stone for future integration
Source: BMC Syst Biol. 2011 Oct 14;5:165. doi: 10.1186/1752-0509-5-165 (PMC3271347; doi:10.1186/1752-0509-5-165)
Supplement: Additional file 11 — Metabolite counts per database for the comparison of core metabolic processes. For the metabolites of the core metabolic processes in each of the five pathway databases the percentage of metabolites without a chemical formula and the percentage of metabolites without an identifier is indicated. Furthermore, for each pathway database the percentage of metabolites linked to a particular metabolite database (KEGG Compound, KEGG Glycan, ChEBI, PubChem Compound, and CAS) is indicated. We also included the instances of metabolite classes for HumanCyc and members of sets for Reactome, see Materials and Methods. [file 1752-0509-5-165-S11.PDF]

Additional file 11 – Metabolite counts per database for the comparison of core metabolic processes

Database	total number of metabolites	% of metabolites without a chemical formula	% of metabolites without identifier	# of metabolites with identifier	% metabolites with				
					KEGG Compound	KEGG Glycan	ChEBI	PubChem Compound	CAS
BiGG	1041	0	21	824	94	5	0	2	64
EHMN	1924	2	38	1195	96	0	62	40	46
HumanCyc	847	6	13	738	88	0	56	87	51
KEGG	1190	2	0	1190	100	3	79	81	50
Reactome	532	22	10	481	92	0	99	58	0

We included the instances of metabolite classes for HumanCyc and members of sets for Reactome, see Materials and Methods. The percentages of the different types of metabolite identifiers are calculated w.r.t. the number of metabolites with at least one identifier. Note that in KEGG each metabolite has at least an identifier from one of its own metabolite databases, KEGG Compound and KEGG Glycan. For 9% of the metabolites there is no other identifier provided.
