# Supplementary material for: Critical assessment of human metabolic pathway databases: a stepping stone for future integration
Source: BMC Syst Biol. 2011 Oct 14;5:165. doi: 10.1186/1752-0509-5-165 (PMC3271347; doi:10.1186/1752-0509-5-165)
Supplement: Additional file 12 — TCA cycle: majority vote. Adapted version of Figure 2 in the main text when retaining only the entities that at least three out of five databases agree on. Reactions occurring in the majority are highlighted. Metabolites are represented by rectangles, genes by rounded rectangles, and EC numbers by parallelograms. Color indicates how many of the five databases include a specific entity. Color of an arrow indicates the number of databases that agree upon an entire reaction, i.e., all its metabolites (except H+ which was matched separately). [file 1752-0509-5-165-S12.PDF]

Additional file 12 – TCA cycle: majority vote

Adapted version of Figure 2 in the main text when retaining only the entities that at least three out of five databases agree on. Reactions occurring in the majority are highlighted. Metabolites are represented by rectangles, genes by rounded rectangles, and EC numbers by parallelograms. Color indicates how many of the five databases include a specific entity. Color of an arrow indicates the number of databases that agree upon an entire reaction, *i.e.*, all its metabolites (except H^+ which was matched separately).

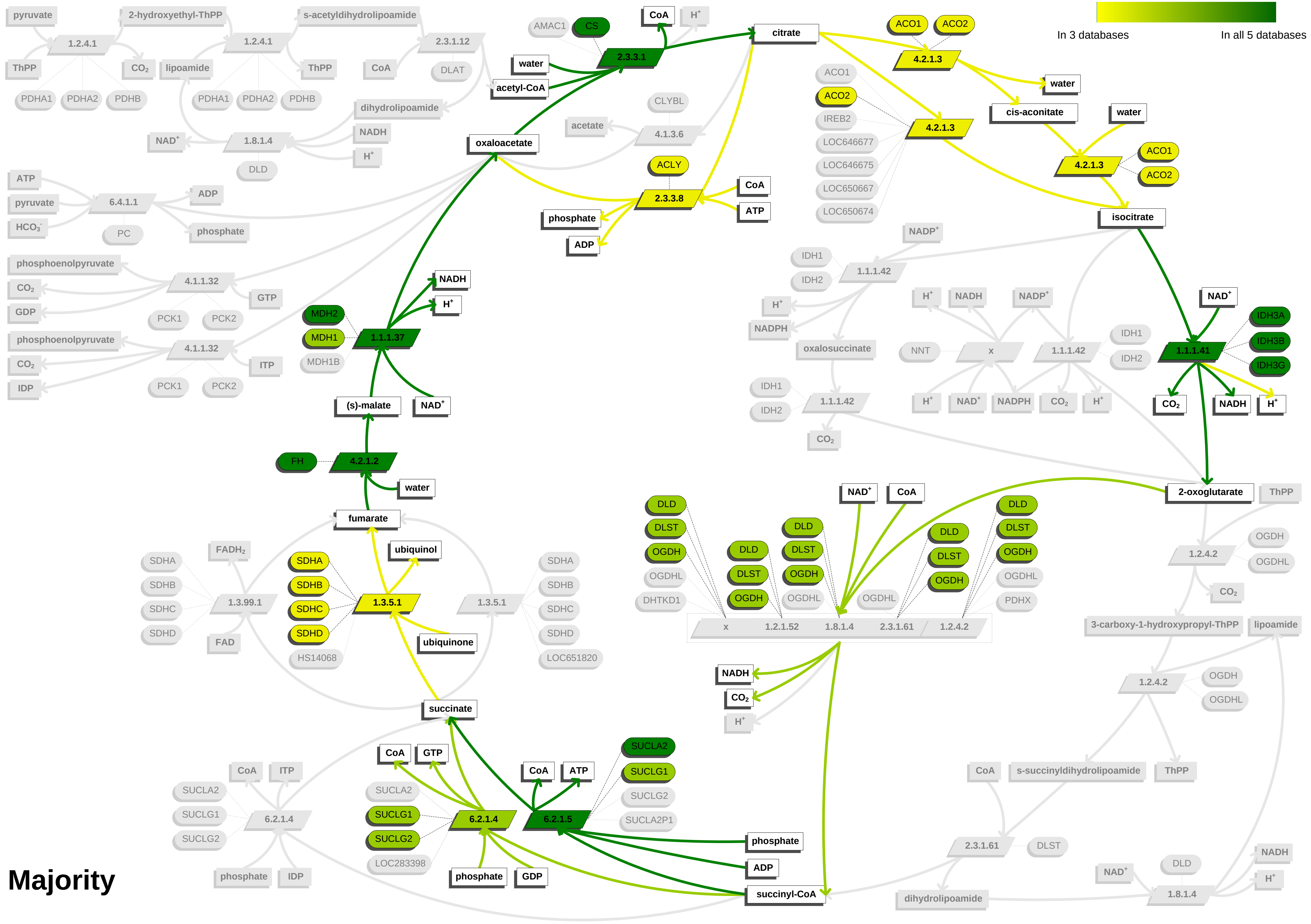
In 3 databases
In all 5 databases
pyruvate
2-hydroxyethyl-ThPP
s-acetyldihydrolipoamide
1.2.4.1
1.2.4.1
2.3.1.12
ThPP
CO_2
lipoamide
ThPP
CoA
DLAT
PDHA1
PDHA2
PDHB
PDHA1
PDHA2
PDHB
dihydrolipoamide
NADH
NAD^+
1.8.1.4
H^+
DLD
ATP
pyruvate
HCO_3^-
6.4.1.1
ADP
PC
phosphate
phosphoenolpyruvate
4.1.1.32
CO_2
GTP
GDP
PCK1
PCK2
phosphoenolpyruvate
4.1.1.32
CO_2
ITP
IDP
PCK1
PCK2
AMAC1
CS
CoA
H^+
2.3.3.1
water
acetyl-CoA
citrate
oxaloacetate
acetate
CLYBL
4.1.3.6
ACLY
2.3.3.8
CoA
ATP
phosphate
ADP
ACO1
ACO2
4.2.1.3
water
ACO1
ACO2
IREB2
LOC646677
LOC646675
LOC650667
LOC650674
4.2.1.3
cis-aconitate
water
4.2.1.3
ACO1
ACO2
isocitrate
$NADP^+$
IDH1
IDH2
1.1.1.42
H^+
NADPH
H^+
NADH
$NADP^+$
oxalosuccinate
NNT
x
1.1.1.42
IDH1
IDH2
H^+
NAD^+
NADPH
CO_2
H^+
IDH1
IDH2
1.1.1.42
CO_2
NAD^+
IDH3A
IDH3B
1.1.1.41
IDH3G
CO_2
NADH
H^+
NADH
H^+
MDH2
MDH1
1.1.1.37
MDH1B
(s)-malate
NAD^+
FH
4.2.1.2
water
fumarate
ubiquinol
$FADH_2$
SDHA
SDHB
SDHC
SDHD
1.3.99.1
FAD
SDHA
SDHB
SDHC
SDHD
HS14068
1.3.5.1
ubiquinone
1.3.5.1
SDHA
SDHB
SDHC
SDHD
LOC651820
succinate
2-oxoglutarate
ThPP
NAD^+
CoA
DLD
DLST
OGDH
OGDHL
DHTKD1
DLD
DLST
OGDH
DLD
DLST
OGDH
OGDHL
OGDHL
DLD
DLST
OGDH
DLD
DLST
OGDH
OGDHL
PDHX
x
1.2.1.52
1.8.1.4
2.3.1.61
1.2.4.2
1.2.4.2
OGDH
OGDHL
CO_2
3-carboxy-1-hydroxypropyl-ThPP
lipoamide
NADH
CO_2
H^+
1.2.4.2
OGDH
OGDHL
CoA
s-succinyldihydrolipoamide
ThPP
2.3.1.61
DLST
dihydrolipoamide
NAD^+
DLD
1.8.1.4
NADH
H^+
CoA
ITP
SUCLA2
SUCLG1
SUCLG2
6.2.1.4
phosphate
IDP
CoA
GTP
SUCLA2
SUCLG1
SUCLG2
LOC283398
6.2.1.4
phosphate
GDP
CoA
ATP
6.2.1.5
SUCLA2
SUCLG1
SUCLG2
SUCLA2P1
phosphate
ADP
succinyl-CoA

Majority
