# Supplementary material for: Critical assessment of human metabolic pathway databases: a stepping stone for future integration
Source: BMC Syst Biol. 2011 Oct 14;5:165. doi: 10.1186/1752-0509-5-165 (PMC3271347; doi:10.1186/1752-0509-5-165)
Supplement: Additional file 14 — Top-level pathways from Reactome (not) considered in the comparison. [file 1752-0509-5-165-S14.PDF]

Additional file 14 – Top-level pathways from Reactome (not) considered in the comparison

Included:

Biological oxidations
Metabolism of amino acids and derivatives
Metabolism of carbohydrates
Metabolism of lipids and lipoproteins
Metabolism of nucleotides
Metabolism of porphyrins
Metabolism of vitamins and cofactors
Pyruvate metabolism and Citric Acid (TCA) cycle
Respiratory electron transport, ATP synthesis by chemiosmotic coupling, and heat production by uncoupling
Transmembrane transport of small molecules

Excluded:

Apoptosis
Axon guidance
Botulinum neurotoxicity
Cell Cycle Checkpoints
Cell Cycle, Mitotic
Cell junction organization
Chromosome Maintenance
Circadian Clock
Diabetes pathways
DNA Repair
DNA Replication
Gene Expression
HIV Infection
Hemostasis
Influenza infection
Integration of energy metabolism
Integrin cell surface interactions
Interactions of the immunoglobulin superfamily (IgSF) member proteins
Meiotic Recombination
Membrane Trafficking
Metabolism of nitric oxide
Metabolism of proteins
Metabolism of RNA
Muscle contraction
mRNA Processing
Myogenesis
Opioid Signalling
Regulation of beta-cell development
Regulatory RNA pathways
Signaling by BMP
Signaling by EGFR
Signaling by FGFR
Signaling by GPCR
Signaling by PDGF
Signaling in Immune system
Signaling in Insulin receptor
Signaling by NGF
Signaling by Notch
Signaling by Rho GTPases
Signaling by TGF beta
Signaling by VEGF
Signaling by Wnt
Synaptic Transmission
Transcription
