# Supplementary material for: Critical assessment of human metabolic pathway databases: a stepping stone for future integration
Source: BMC Syst Biol. 2011 Oct 14;5:165. doi: 10.1186/1752-0509-5-165 (PMC3271347; doi:10.1186/1752-0509-5-165)
Supplement: Additional file 15 — Instantiating reactions containing sets of metabolites. Reactome contains reactions defined in terms of sets of metabolites. For five of such reactions the specific instantiations could not be derived automatically, this document gives two detailed examples. [file 1752-0509-5-165-S15.PDF]

Additional file 15 – Instantiating reactions containing metabolite sets

Reactome contains reactions defined in terms of sets of metabolites. For our comparison we wanted to use the specific reactions that can be derived from these. According to the curator guide of Reactome (http://wiki.reactome.org/index.php/Reactome_Curator_Guide) if a set is used as input for a reaction and another set as output, the annotation is taken to mean that the first member of the input set is converted to the first member of the output set and so on. This indeed works in most cases, except for five cases, including the following two examples.

Example 1

```
(choloyl-CoA, chenodeoxycholoyl-CoA) + (glycine, taurine)
                                    →
(glycocholate, glycochenodeoxycholate, taurocholate, taurochenodeoxycholate)
+ CoA
```

If we would take the first member of each set and do the same for the second member, this would give us:

```
choloyl-CoA + glycine → glycocholate + CoA                           (1)
chenodeoxycholoyl-CoA + taurine → glycochenodeoxycholate + CoA       (2)
```

This leaves us with the two last members of the set in the output; assuming we should recycle the members of the sets in the input of the left hand side of the reaction:

```
choloyl-CoA + glycine → taurocholate + CoA                           (3)
chenodeoxycholoyl-CoA + taurine → taurochenodeoxycholate + CoA       (4)
```

Reaction 1 and 4 are indeed correct, but reaction 2 and 3 are not.

Another option would be to make all possible combinations between the sets, which would give 16 reactions of which only 4 are correct:

```
choloyl-CoA + glycine → glycocholate + CoA
choloyl-CoA + taurine → taurocholate + CoA
chenodeoxycholoyl-CoA + glycine → glycochenodeoxycholate + CoA
chenodeoxycholoyl-CoA + taurine → taurochenodeoxycholate + CoA
```

Example 2

(TMP, uridine 5' monophosphate, 2'-deoxyuridine 5' monophosphate, uridine 2' monophosphate, uridine 3' monophosphate) + H_2O

→

(thymidine, uridine, deoxyuridine) + orthophosphate

In this example there are five metabolites in the set at the left hand side of the reaction and only three metabolites at the right hand side, which again makes it impossible to match the first member of one set with the first of the other, and so on.
